# Conservation and divergence of the p53 gene regulatory network between mice and humans

**DOI:** 10.1038/s41388-019-0706-9

**Published:** 2019-02-01

**Authors:** Martin Fischer

**Affiliations:** 1 Computational Biology Group, Leibniz Institute on Aging – Fritz Lipmann Institute (FLI), 07745 Jena, Germany; 2 Molecular Oncology Group, Medical School, University of Leipzig, 04103 Leipzig, Germany

**Keywords:** Transcription, Gene regulation

## Abstract

Understanding the p53 tumor suppressor pathway remains crucial for the design of anticancer strategies. Studies in human tumors and mouse models help to unravel the molecular mechanisms that underlie the p53 signaling pathway. Yet, the p53 gene regulatory network (GRN) is not the same in mice and humans. The comparison of the regulatory networks of p53 in mice and humans reveals that gene up- and down-regulation by p53 are distinctly affected during evolution. Importantly, gene up-regulation by p53 underwent more rapid evolution and gene down-regulation has been evolutionarily constrained. This difference stems from the two major mechanisms employed by p53 to regulate gene expression: up-regulation through direct p53 target gene binding and indirect down-regulation through the p53-p21-DREAM pathway. More than 1000 genes have been identified to differ in their p53-dependent expression between mice and humans. Analysis of p53 gene expression profiles and p53 binding data reveal that turnover of p53 binding sites is the major mechanism underlying extensive variation in p53-dependent gene up-regulation. Only a core set of high-confidence genes appears to be directly regulated by p53 in both species. In contrast to up-regulation, p53-induced down-regulation is well conserved between mice and humans and controls cell cycle genes. Here a curated data set is provided that extends the previously established web-atlas at www.targetgenereg.org to assess the p53 response of any human gene of interest and its mouse ortholog. Taken together, the analysis reveals a limited translation potential from mouse models to humans for the p53 GRN.

## Introduction

The tumor suppressor p53 functions as a transcription factor (TF) that regulates a plethora of genes, either by direct or indirect mechanisms [1, 2]. While p53 directly up-regulates target genes, down-regulation of target genes is largely mediated indirectly through the transcription repressor complex DREAM (dimerization partner, RB-like, E2F and multi-vulval class B) [3, 4]. p53 employs p21, encoded by the direct p53 target gene *CDKN1A*, to stabilize the DREAM complex, which specifically down-regulates cell cycle genes [5, 6]. Through its direct target genes p53 regulates key cellular processes such as proliferation, apoptosis and metabolism to suppress tumorigenesis [1].

Despite the frequent use of mouse models for the study of p53 [7–9], there has been a lack of information that address common and divergent parts of the p53 gene regulatory network (GRN) between mice and humans. There is evidence that gene expression and underlying regulatory programs have substantially diverged between mice and humans and that some groups of genes and regulatory elements have undergone more rapid evolution than others [10]. On average ~44% of regulatory TF to gene associations have been found to be conserved between mice and humans [11]. The p53 tumor suppressor function is conserved, and, similar to most TFs [11–13], p53 homologs from multiple species have been shown to contain a conserved DNA binding domain that can specifically trans-activate a common binding motif: the p53 response element (p53RE) [14]. Yet, gene regulation has been reported to differ for at least some of its targets. For example, the proteins encoded by the human direct p53 target genes *GADD45A* and *RRM2B* were shown to induce G2/M cell cycle arrest [15] and to supply precursors for DNA repair [16], respectively. Their mouse orthologs, however, are not regulated by p53 [17]. While DNA sequences that recruit TFs and contribute to target gene regulation often display phylogenetic conservation [18], comparison of several p53REs revealed only limited conservation across species [17, 19]. A recent study revealed that p53 oscillates faster in mouse and rat cells than in cells from humans, monkeys or dogs [20]. It remained elusive, however, to what extent the difference in p53 oscillation results in alterations of the p53 GRN [20].

The recent expansion of high-throughput data sets enables comprehensive comparison of the p53 GRN between mice and humans and identification of the mechanisms that underlie the inclusion or exclusion of target genes during evolution. Because results typically vary from one study to the next, a recently developed meta-analysis approach has been used to synthesize data across studies [4]. By combining multiple expression profiling data sets with chromatin binding sites, high-confidence targets are identified that are more likely to be regulated by any given transcription factor. The previously established web-based atlas on p53-dependent regulation of human genes (www.targetgenereg.org) [4] is extended by a ranked list of p53-regulated genes in the mouse genome. The comparison of ranked lists of mouse and human p53-regulated genes provides a comprehensive overview of conserved and species-specific p53-regulated genes and enables identification of the mechanisms that shape the p53 GRN during evolution.

## Results

### Transcriptional landscape of p53-regulated genes in the mouse genome

In recent years multiple genome-wide p53 gene expression data sets have become available for mice. Because it is generally agreed that gene expression data from different experimental platforms are not directly comparable, instead the step-wise meta-analysis approach was used, which was employed recently to analyze the p53 GRN in human cells [4]. Analyzing the p53 GRN in mice based on the same approach allows direct comparison of the orthologous networks.

**Fig. 1:**
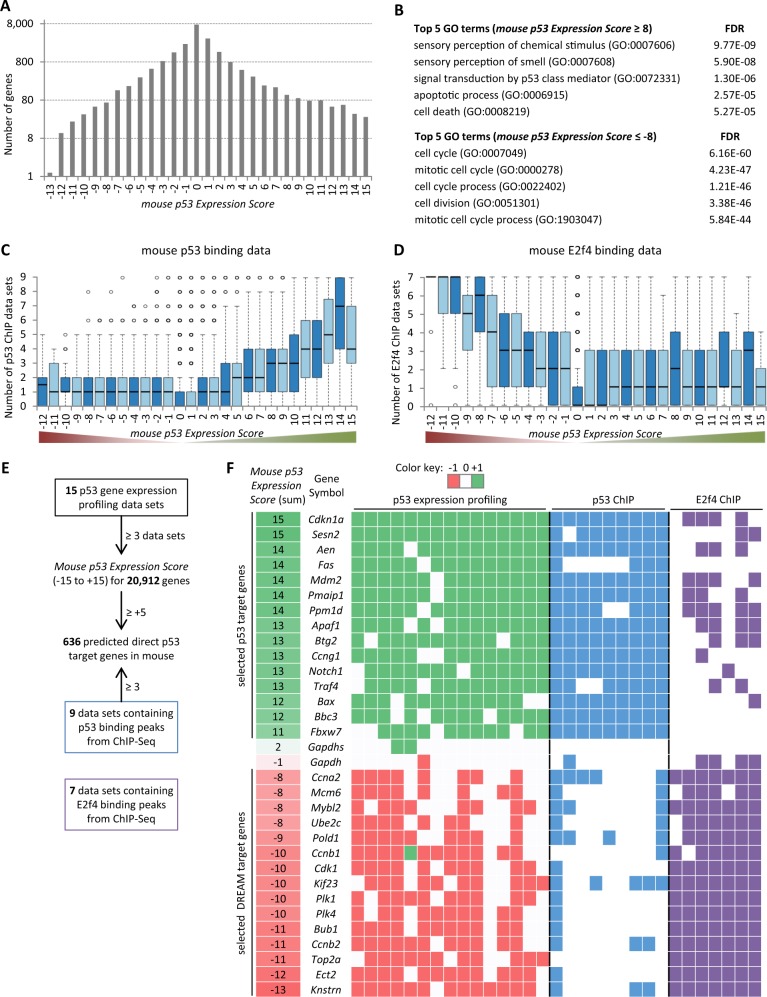
Meta-analysis of p53-dependent gene expression in the mouse genome. **a** The number of genes is displayed that is found in each of the 29 *mouse p53 Expression Score* groups. For further analyses group ‘−13’ was added to group ‘−12’. **b** Top five biological process gene ontology (BP GO) terms with their false discovery rate (FDR) enriched at genes that are found commonly up- (*n* = 534) or down-regulated (*n* = 191) in at least half of the 15 data sets. **c** Boxplot displaying the number of chromatin immunoprecipitation (ChIP) data sets that find a gene to be bound by p53 within 5 kb of their transcriptional start site (TSS) across the 28 *mouse p53 Expression Score* groups. **d** Boxplot displaying the number of ChIP data sets that find a gene to be bound by E2f4 within 1 kb of their TSS across the 28 *mouse p53 Expression Score* groups. **e** Flow chart for mouse data integration. **f** A heatmap displaying the regulation of 15 well-established p53 or DREAM target genes across the 15 data sets on p53-dependent gene regulation, 9 data sets on p53 binding and 7 E2f4 binding data sets. *Gapdh* and *Gapdhs* serve as negative controls

From 10 genome-wide studies [21–30], 15 gene expression profiling data sets were integrated (Supplementary Figures S1 and S2) that have been derived from mouse embryonic fibroblasts (MEFs; *n* = 11), mouse embryonic stem cells (mESCs; *n* = 2), mouse thymus cells (*n* = 1) or mouse B cells (*n* = 1) stimulated with doxorubicin (*n* = 7), sip53 (*n* = 3), ionizing radiation (*n* = 2), ultraviolet (*n* = 1), KRAS (*n* = 1) or p53 knock-in (*n* = 1). Synthesizing data across cell types and treatments enables identification of genes that are commonly regulated by p53, as shown previously [4]. For 20,912 genes present in at least three data sets, a *mouse p53 Expression Score* was calculated as the number of data sets that find the gene to be significantly up-regulated minus the number of data sets that find the gene to be down-regulated when p53 is active. This resulted in 29 gene groups because no gene was identified as down-regulated in 14 or all 15 data sets (Fig. 1a and Supplementary Table S1), and given that the gene group ‘−13’ contained only one gene, it was included in group ‘−12’ for further analyses. Similar to results from the individual studies, gene ontology (GO) terms associated with p53 signaling and apoptosis are enriched for commonly up-regulated genes, and GO terms associated with cell cycle and mitosis are enriched for commonly down-regulated genes (Fig. 1b and Supplementary Table S3).

Because p53 and DREAM are known to up- and down-regulate most p53-dependently regulated genes, respectively, mouse p53 and E2f4 binding data from genome-wide chromatin immunoprecipitation (ChIP) experiments was integrated. Binding of the key DREAM complex component E2f4 [5] served as a proxy for DREAM binding because no mouse binding data were available for additional DREAM components. The 20,912 mouse genes were assigned a p53 and E2f4 ChIP score based on the number of data sets that identify a p53 and E2f4 ChIP peak in proximity of the transcriptional start site (TSS), respectively (Supplementary Table S4). Comparison of the mouse p53 binding score and the *mouse p53 Expression Score* from all genes shows that high-confidence p53 binding correlates with target gene up-regulation (Fig. 1c). In contrast, E2f4 binding correlates with down-regulation of target genes in response to p53 signaling (Fig. 1d). These results are consistent with human data that found p53 up-regulated genes to be enriched for p53 binding and p53 down-regulated genes enriched for DREAM binding [3, 4].

By integrating the p53 binding and p53-dependent expression profiling data sets, a list of potential direct p53 target genes in the mouse genome was generated with a threshold for *mouse p53 Expression Score* of ≥5 and p53 binding within ±5 kb of the TSS in at least three of the nine ChIP data sets. These thresholds ensure that p53 regulation and binding was observed in data sets from at least two different studies. These criteria were passed by 636 genes including many well-known direct p53 target genes (Fig. 1f and Supplementary Table S5).

To illustrate the utility of the integrative approach, 15 genes known to be direct p53 targets [1] (Supplementary Table S5) and 15 previously published DREAM targets [4, 31–36] were selected and their regulation was examined across all expression profiling data sets (*n* = 15), p53 binding (*n* = 9) and E2f4 binding data sets (*n* = 7; Fig. 1f). Identification of the well-established target genes demonstrates the ability of this approach to identify *bona fide* candidates. Notably, two p53 ChIP-seq data set appear to be rather noisy and five data sets from four studies identify only a small number of p53 down-regulated genes (Fig. 1f and Supplementary Figures S1 and S2).

Together, the step-wise meta-analysis approach captured information from many of the recently reported data sets to generate a mouse p53 GRN that can be compared to the human p53 GRN, which was established recently using the same approach [4].

### Gene down-regulation is more similar than up-regulation by p53 between mice and humans

**Fig. 2:**
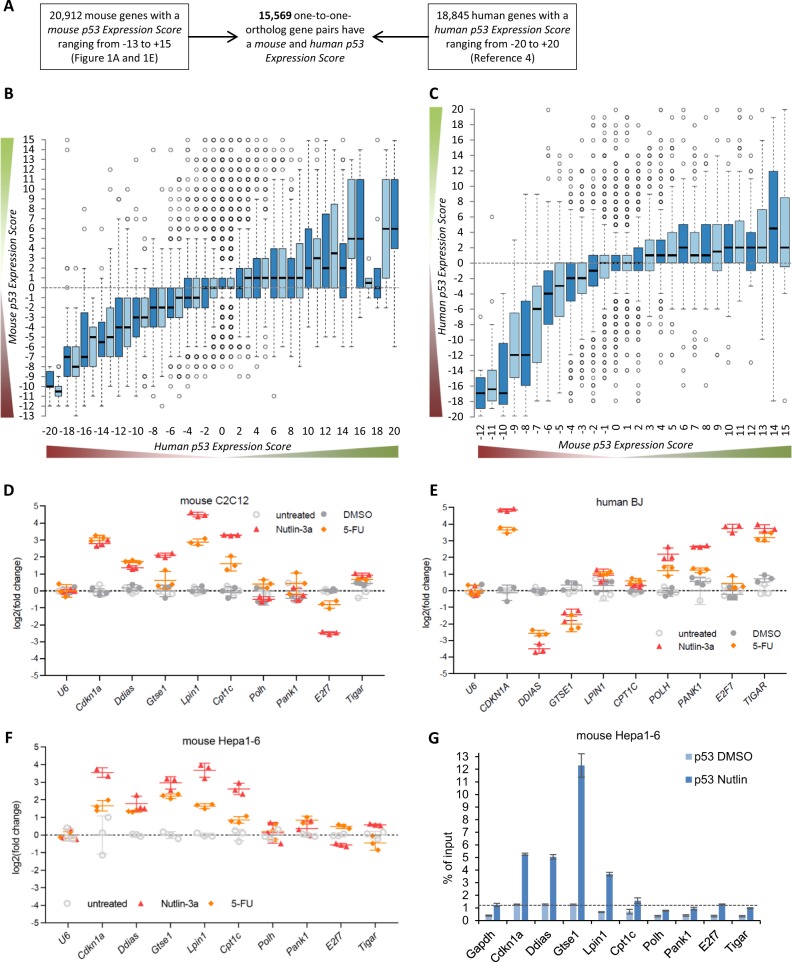
Gene down-regulation is more similar than up-regulation by p53 between mice and humans. **a** Flow chart for the identification of 15,569 one-to-one orthologous gene pairs used for further analysis. **b** The *mouse p53 Expression Score* (Supplementary Table S1) compared to the previously published *p53 Expression Score* generated from human data [4] for 15,569 one-to-one orthologs and **c** vice versa the *human p53 Expression Score* compared to the *mouse p53 Expression Score* (Supplementary Table S6). The log2-fold change of mRNA expression from treated compared to untreated **d** mouse C2C12, **e** human BJ-hTERT and **f** mouse Hepa1-6 cells is displayed. Cells were treated with Nutlin-3a or 5-FU for 24 h. Untreated cells and cells treated with dimethyl sulfoxide (DMSO) served as controls. *U6* expression served as negative control for p53 response and was used for normalization, while *Cdkn1a*/*CDKN1A* was employed as positive control. For comparison, values were normalized to measurements from untreated cells. **g** Chromatin immunoprecipitation – real-time polymerase chain reaction (ChIP-qPCR) results from p53 ChIP in DMSO- and Nutlin-treated Hepa1-6 cells. p53 binding to *Gapdh* and *Cdkn1a* served as negative and positive control, respectively. One representative out of ≥3 biological replicates is displayed with three technical replicates. Means and s.d. are displayed

To identify common and distinct p53-regulated genes in mice and humans, the *mouse p53 Expression Score* (Supplementary Table S1) was compared to the previously published *p53 Expression Score* generated from human data [4]. Both the *mouse* and the *human p53 Expression Score* have been shown to correctly identify *bona fide* p53-regulated genes. A total set of 15,569 ortholog gene pairs was identified that includes all protein-coding genes for which exactly one mouse gene corresponds to exactly one human ortholog (one-to-one orthologs) and for which both were assigned a *p53 Expression Score* (Fig. 2a and Supplementary Table S6).

Comparing the mouse and human *p53 Expression Scores* for all 15,569 gene pairs revealed a better correlation in p53-dependent gene down-regulation (Spearman's *r* 0.336 and 0.344) than in gene up-regulation by p53 (Spearman's *r* 0.120 and 0.186) (Fig. 2b, c). Variation between *mouse* and *human p53 Expression Scores* of multiple ortholog gene pairs is consistent with their p53-dependent regulation in previously published literature. For example, mouse *Gadd45a*, *Rrm2b* and *Ddb2* were assigned *mouse p53 Expression Scores* of ‘0’, ‘2’ and ‘−2’, respectively, and their human orthologs *GADD45A*, *RRM2B* and *DDB2* display *human p53 Expression Scores* of ‘16’, ‘18’ and ‘18’, respectively (Supplementary Table S6). These data are consistent with reports describing human *GADD45A*, *RRM2B* and *DDB2* as being directly up-regulated by p53 [15, 16, 37], while their mouse orthologs are not regulated by p53 [17, 37]. Consistent with the previous observation that mouse *Polk* but not human *POLK* is directly up-regulated by p53 [38], human *POLK* displays a *human p53 Expression Score* of ‘−4’ and mouse *Polk* displays a *mouse p53 Expression Score* of ‘14’. An exceptional example is human *PSRC1* (also known as *DDA3*), which encodes for a protein that functions in spindle assembly during mitosis and is down-regulated by p53 in humans [39]. The transcriptional repressor complex DREAM binds human *PSRC1* and most likely mediates its down-regulation in response to p53 [4]. Mouse *Psrc1*, however, is directly up-regulated by p53 [40]. Consistent with these reports, human *PSRC1* was assigned a *human p53 Expression Score* of ‘−18’ and mouse *Psrc1* displays a *mouse p53 Expression Score* of ‘15’. These examples provide evidence that the integrative approach employed here correctly identifies differences in p53-dependent gene regulation between mice and humans that have been reported earlier.

To generate a global list of all one-to-one ortholog genes that most likely differ in their p53-dependent regulation between mice and humans, genes with an absolute *p53 Expression Score* ≥5 were filtered for those that display an absolute difference between the *mouse* and the *human p53 Expression Score* of ≥8, and for which less than three data sets supported their regulation in the other species. A total set of 1010 genes passed these criteria (Supplementary Table S7). Genes previously unknown to have a species-specific p53 regulation include the cell cycle genes *Ddias* [41] and *Gtse1* [42] that are up-regulated by p53 in mice, but down-regulated in humans (Fig. 2d–f, Supplementary Table S7). In contrast, p53 directly up-regulates human *E2F7* [43], but leads to down-regulation of its mouse ortholog. Moreover, p53 does not up-regulate the mouse orthologs of the human direct p53 target genes *POLH* [44], *PANK1* [45] and *TIGAR* [46]. Similarly, the mouse direct p53 target genes *Lpin1* [47] and *Cpt1c* [48] are not up-regulated by p53 in humans (Fig. 2d–f, Supplementary Table S7). Together, these results demonstrate the ability of the meta-analysis approach to identify common and distinct p53-regulated genes in mice and humans.

When mouse models are employed for the study of p53, it is important to know what biological processes are commonly regulated by p53 and what biological pathways diverged in their p53 response during evolution. Genes commonly up-regulated by p53 in mice and humans are enriched for GO terms associated with apoptosis and cell cycle checkpoints (Supplementary Table S3). In contrast, the 1010 genes that differ in their p53 response between mice and humans are enriched for GO terms related to DNA damage response, DNA metabolism and also to cell cycle regulation. These findings are consistent with the previous finding that several genes involved in DNA metabolism display almost no conservation in their p53 response between mice and humans [17]. Moreover, although DNA damage response and cell cycle regulation is commonly enriched among p53 up-regulated genes in mice and humans, the findings suggest that a substantial number of genes involved in these processes differs in their p53 response between mice and humans. Function in apoptosis regulation, however, is commonly enriched among genes up-regulated by p53 in mice and humans and their p53 response appears not to be subjected to much variation.

### Evolutionary variation in p53-dependent gene up-regulation relates to different p53 binding profiles

**Fig. 3:**
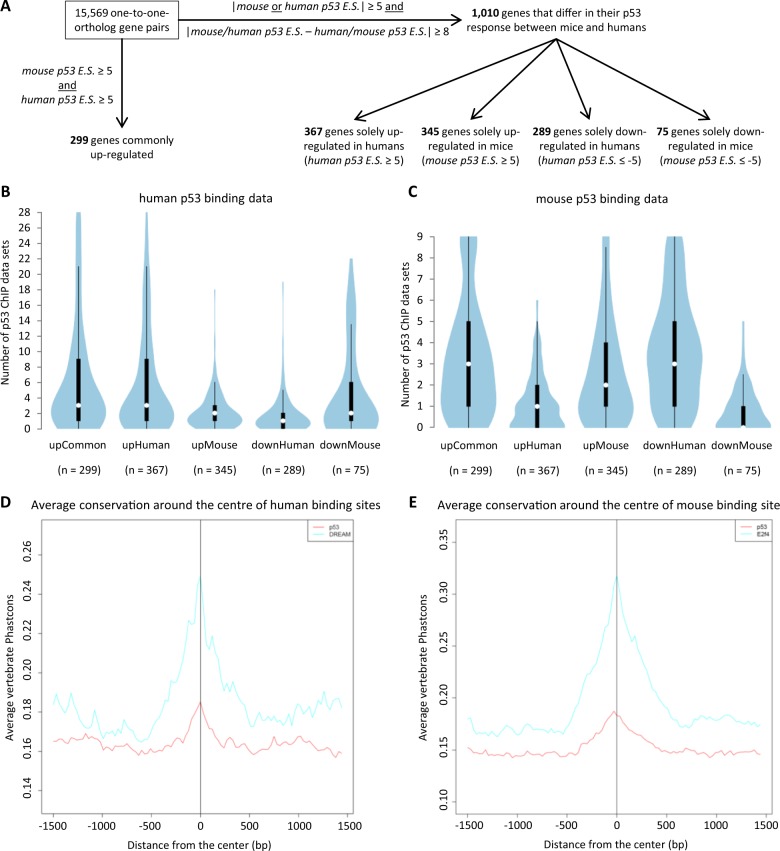
Evolutionary variation in p53-dependent up-regulation relates to different p53 binding profiles and low conservation of DNA binding sites. **a** Flow chart for the selection of gene groups that display distinct p53-dependent regulation between mice and humans. *p53 E.S*. *p53 Expression Score*. Violin plots display the p53 binding scores that were associated with these gene groups in **b** humans and **c** mice. White circles show the medians; box limits indicate the 25th and 75th percentiles; whiskers extend 1.5 times the interquartile range from the 25th and 75th percentiles; polygons represent density estimates of data and extend to extreme values. Conservation plots displaying the average vertebrate PhastCons score for **d** 7635 human p53 (red) and 2565 DREAM (cyan) binding sites supported by at least 5 out of 28 and 4 out of 9 data sets, respectively, and **e** 9346 mouse p53 (red) and 3933 E2f4 (cyan) binding sites supported by at least 4 out of 7 data sets

The surprisingly little conservation of p53-dependent gene up-regulation between mice and humans led to the question of whether this variation is related to differences in direct p53 target gene binding. Thus, several p53 binding profiles were collected to compare mouse (*n* = 9) and human (*n* = 28) p53 binding (Supplementary Tables S4 and S8). First, the p53 binding data were examined for the genes that have been identified above to have a species-specific p53 response (Fig. 2d–f): *POLH*, *E2F7*, *TIGAR* and *PANK1* are up-regulated solely in human and p53 binding near their TSS has been identified in multiple data sets from humans but not from mice. In contrast, *Gtse1*, *Ddias*, *Lpin1* and *Cpt1c* are up-regulated solely in mice and p53 binding near their TSS has been identified in multiple data sets from mice but not from humans (Supplementary Figures S6-13). These findings have also been confirmed in the mouse epithelial cell line Hepa1-6 through ChIP-qPCR (Fig. 2g). To gain a more comprehensive view, the p53 binding scores were compared for selected gene groups that display distinct regulation patterns (Fig. 3a). One group (*n* = 299) comprises genes commonly up-regulated by p53. Additionally, subgroups were generated out of the 1010 genes that appear to differ strongly in their p53-dependent regulation between mice and humans (Supplementary Table S7): 367 genes were solely up-regulated in humans; 345 genes were solely up-regulated in mice; 289 genes were solely down-regulated in humans; and 75 genes were solely down-regulated in mice (Fig. 3a and Supplementary Table S7). As expected, the data show that p53 binding near the TSS in mice and humans is associated with p53-dependent up-regulation in humans as well as in mice (Fig. 3b, c). More importantly, the analysis reveals that genes up-regulated by p53 in humans but not in mice are also associated with direct p53 target gene binding solely in humans. The same is true vice versa for genes up-regulated by p53 in mice but not in humans. For a number of genes that are down-regulated solely in mice or humans, the data also indicate that loss of down-regulation coincided with gain of p53 binding (Fig. 3b, c). Together, these results reveal that evolutionary variation in p53-dependent gene up-regulation relate to different p53 binding profiles.

### p53REs are frequently altered and only a small number of p53 binding sites is conserved during evolution

In contrast to gene up-regulation by p53, gene down-regulation appears to be well conserved between mice and humans (Fig. 2b, c). These data are consistent with p53-dependent gene down-regulation being mediated indirectly through the DREAM complex [3, 4, 32]: binding profiles of the DREAM component E2F4 have been shown to be retained between mice and humans [49]. Moreover, human DREAM and mouse E2f4 binding sites display high evolutionary conservation scores for their underlying DNA, while human and mouse p53 binding sites display low conservation scores (Fig. 3d, e).

**Fig. 4:**
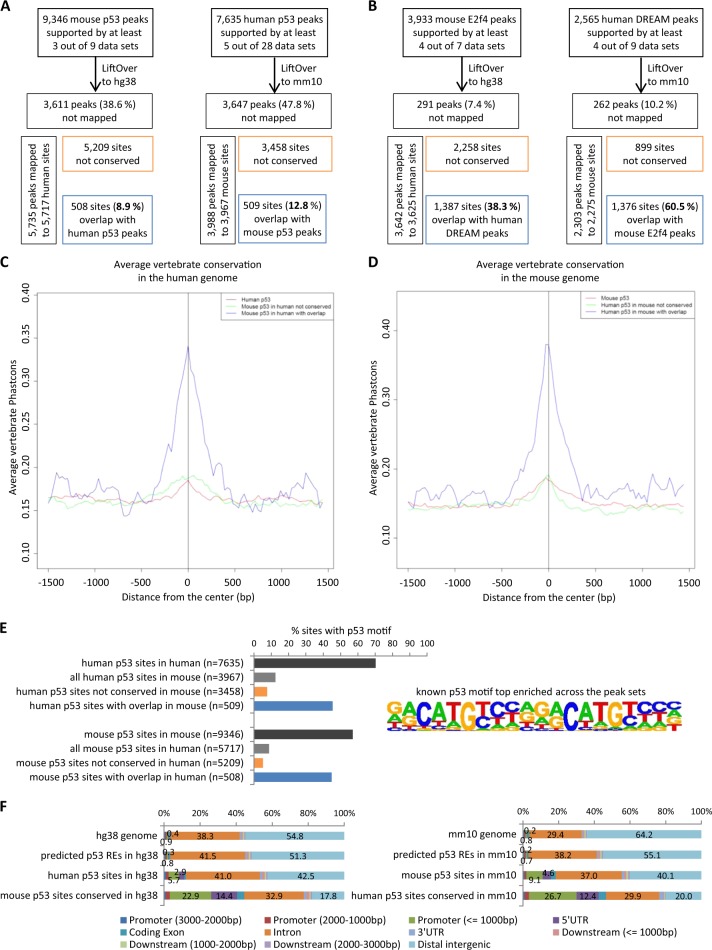
Changes in p53 binding profiles relate to alterations in p53REs. Mouse and human peak sets for **a** p53 and **b** DREAM/E2f4 were mapped to the other genome and overlapping and non-overlapping binding sites were identified. Conservation plots displaying the average vertebrate PhastCons score for **c** 7635 human p53 peaks (red), 5209 mouse p53 peaks mapped to the human genome that are not conserved for p53 binding (green) and 508 mouse p53 peaks mapped to the human genome that overlap with human p53 peaks (blue) and **d** 9346 mouse p53 peaks (red), 3458 human p53 peaks mapped to the mouse genome that are not conserved for p53 binding (green) and 509 human p53 peaks mapped to the mouse genome that overlap with mouse p53 peaks (blue). **e** Quantification of ‘known’ p53 motif occurrence by HOMER in the peak sets determined in (**a**). **f** Enrichment of p53 binding sites at genome features compared to the expected genomic distribution as identified by CEAS

To quantify the conservation of binding sites, mouse p53 and E2f4 binding sites were mapped to the human genome and human p53 and DREAM binding sites were mapped to the mouse genome (Fig. 4a). Strikingly, only 8.9 to 12.8% of the sites present in both genomes appear to be conserved for p53 binding. In contrast to p53, 38.3% of the mouse E2f4 binding sites and 60.5% of the human DREAM binding sites overlap with human DREAM and mouse E2f4 binding sites, respectively, for regions that are present in both genomes (Fig. 4b). Notably, sites that are occupied by p53 in both the mouse and the human genome display a markedly higher vertebrate conservation score compared to sites that are not commonly bound or compared to all sites (Fig. 4c, d), which indicates that these sites may be conserved in multiple vertebrates in addition to mice and humans.

Given the low conservation of the DNA underlying most p53 binding sites and the little overlap between mouse and human p53 binding sites, it is likely that alterations in the p53REs occurred between mice and humans. Identification of binding sites that display a known p53RE using HOMER revealed that the majority of mouse (57.1% of 9346) and human (70.3% of 7635) high-confidence p53 binding sites contain a canonical p53RE (Fig. 4e). Consistent with recent evolutionary analyses of other TFs showing that most *cis*-regulatory elements are not retained between mice and humans [10, 11, 50], only a fraction of the p53REs are conserved between mice and humans (Fig. 4e). Sites that are bound by p53 solely in humans but not in mice unlikely contain p53REs in mice and sites that are bound by p53 solely in mice but not in humans unlikely contain p53REs in humans. Concordantly, p53REs bound by p53 in human *PANK1*, *E2F7* and *TIGAR* are conserved in primates and some glires, but altered in murinae (Supplementary Figure S14). In addition, p53REs bound by p53 in mouse *Ddias*, *Gtse1*, *Lpin1* and *Cpt1c* are conserved in murinae, but altered in some other glires and in primates. Thus, loss and gain of p53 binding sites relates to loss and gain of p53REs, respectively.

Similar to the higher number of p53 ChIP-seq binding sites in mice (Fig. 4a), there are also more potential p53REs, as predicted using HOMER, in the mouse genome (*n* = 124,313) as compared to the human genome (*n* = 98,553). Binding of mouse and human p53 is ~5 to 10-fold enriched at gene promoters and 5’untranslated region (UTR) compared to the general genomic distribution (Fig. 4f), which is consistent with data showing that p53 up-regulates most of its direct targets through binding within 2.5 kb from the TSS [4, 51]. Predicted p53REs, however, are rather evenly distributed across the mouse and human genomes, which, together with the much lower number of p53 binding sites, indicates that p53REs alone are not sufficient to confer p53 binding. More importantly, sites conserved for p53 binding between mice and humans are another ~3-fold more likely to be located in gene promoters (<1 kb from the TSS) and 5’UTR compared to all p53 binding sites. Consistent with data from other TFs [12], evolutionary turnover of p53 binding sites is also most pronounced in distal intergenic regions.

In summary, the analyses reveal that evolutionary turnover of p53REs leads to extensive variation in p53 binding profiles that ultimately result in different p53-dependent gene up-regulation between mice and humans. Only a small number of p53 binding sites preferably located in promoters and 5’UTR appears to be conserved throughout vertebrate evolution. In contrast, indirect gene down-regulation by p53 mediated through p21 and the E2f4-containing DREAM complex appears to be evolutionarily well conserved.

### A small core set of 86 common direct p53 target genes in mice and humans is particularly enriched for function in apoptosis

**Fig. 5:**
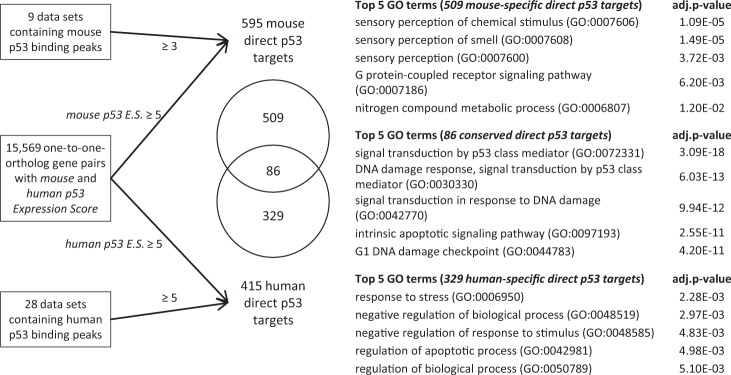
Functions of common and species-specific direct p53 target genes. A flow chart displays the identification of direct p53 target genes in the mouse and the human genome and the top BP GO terms enriched at 509 mouse-specific, 329 human-specific and 86 common direct p53 target genes with their FDR

The genes that are sustained as direct p53 targets during evolution may be particularly important targets for p53-mediated tumor suppression. A total of 636 genes have been identified as potential direct p53 targets in the mouse genome (Fig. 1e and Supplementary Table S5), and 595 of these genes have one-to-one orthologs in the human genome (Supplementary Table S6). To identify human genes that are also potential direct p53 target genes, a threshold was applied for *human p53 Expression Score* of ≥5 and p53 binding within ±5 kb of the TSS in at least 5 of the 28 ChIP data sets. These criteria were passed by 451 genes and 415 of these have a one-to-one ortholog in the mouse genome. Further analysis focused only on the one-to-one otholog gene pairs to ensure direct comparability. The criteria for a direct p53 target gene in both mice and humans were passed by 86 one-to-one ortholog gene pairs (Fig. 5 and Table 1). Thus, only 14–21% of the direct p53 target genes with one-to-one orthologs identified in mouse and human are common, which is considerably lower than the ~44% conserved TF to gene connections that were identified on average between mice and humans [11]. The conserved direct p53 target genes can be grouped into p53 binding to a site that overlaps in mice and humans and p53 binding to species-specific sites (non-overlapping) near the TSS of the orthologous target gene (Supplementary Figure S15). For most TFs the latter group reportedly accounts for >40% of conserved TF to target gene associations [11]. Analysis of the p53 network revealed that 67% (58 out of 86) of direct target genes are evolutionarily sustained through p53 binding to overlapping sites (Supplementary Figure S15) and p53 binding to species-specific sites accounts only for the minor part (28 out of 86) of conserved direct p53 target genes.

**Table 1:** The 86 direct p53 target genes commonly predicted in mice and humans

*ABCC5*	***CSNK1G1***	*GDF15*	***MRPL39***	*PRKD2*	*TMBIM1*
*ACER2*	*DDR1*	*GRHL3*	*NINJ1*	***RAP2B***	***TMEM63B***
***AEN***	***DGKA***	***GSS***	***NOTCH1***	*RASL11A*	***TP53***
*ALDH4A1*	***DYRK3***	*HAGH*	***NTN4***	*RHOD*	***TP53INP1***
***APAF1***	***EDA2R***	*HEXIM1*	***PARD6G***	***RIN1***	***TRAF4***
*ATP6V1H*	***EI24***	***HRAS***	*PERP*	*RIN2*	***TRIAP1***
***BAX***	***ELL***	***IKBIP***	***PHLDA3***	***RPS27L***	***TRIM32***
***BBC3***	***EPHA2***	***ITPKC***	***PIDD1***	***SAC3D*** *1*	***TSKU***
***BLOC1S2***	*EPHX1*	*KCTD11*	*PLAU*	***SESN2***	***ZMAT3***
***BMP7***	***FAM212*** *B*	***KLHDC7A***	***PLK2***	***SLC12A4***	***ZNF219***
***BTG2***	*FAS*	***LIF***	***PLTP***	*SLC4A11*	*ZNF385A*
***CCNG1***	***FBXW7***	*LTBP2*	***PLXNB2***	*SPRYD4*	
***CDKN1A***	***FDXR***	***MDM2***	***PMAIP1***	***SULF2***	
***CHST14***	***FITM2***	*MFGE8*	*PML*	***SUSD6***	
***CPEB2***	*FRMD8*	*MRI1*	***PPM1D***	***SYTL1***	

In all, 58 genes are associated with a conserved binding site (in bold) and 28 genes display different p53 binding sites in mice and humans

Notably, almost half of the 86 common direct p53 target genes and their encoding proteins have not yet been studied for their function in the p53 response [1]. The genes are enriched for GO terms associated with signal transduction by p53, apoptosis and cell death (Fig. 5 and Supplementary Table S3). These results stand in contrast to an earlier analysis of 83 p53REs where apoptosis-regulating p53REs displayed very little conservation [19]. Results from the comprehensive data analysis here, however, are consistent with reports that find apoptosis to be the best conserved function between p53 and its ancestral homologs [52]. Notably, mouse- and human-specific direct p53 target genes were not enriched for known p53 tumor suppressor functions (Fig. 5 and Supplementary Table S3).

Together, the results show that only a small set of genes are conserved for direct p53 control, which largely relates to the small number of p53 binding events that is shared. This small set of common direct p53 target genes, however, is particularly enriched for the core p53 function apoptosis.

## Discussion

Although mouse models are frequently used for the study of p53, a comprehensive assessment of the conserved and divergent parts of the p53 GRN between mice and humans has been missing. An increasing number of reports has identified differences in the p53 pathway between mouse and human and concerns begin to emerge regarding the applicability of mouse models for the study of p53. Here, the expansion of available high-throughput data sets was utilized to generate ranked lists of p53-regulated genes in the mouse genome. Comparing the ranked lists of p53-regulated genes from mouse to previously generated lists from human data [4] reveals a more complete picture of the p53 GRN. The curated mouse data have been made available through the previously established web-atlas at www.targetgenereg.org [4] for all 15,569 one-to-one orthologs in humans.

### Limitations of the meta-analysis

The findings of the meta-analysis are based on the data provided by the underlying data sets. Taking cell types into account, the mouse p53 GRN is biased towards MEF and mESC, while the human p53 GRN is based on more diverse cell types. Taking treatments into account, the mouse p53 GRN is biased towards genes regulated by doxorubicin, while the human p53 GRN is biased more strongly towards genes regulated by Nutlin in addition to doxorubicin. Taking mapping into account, differences caused by different repeat regions in the genomes could confound the results. Although many p53 responsive genes are regulated by p53 robustly across multiple cell types and treatments, insights from the meta-analysis may be limited when investigating treatment-cell type combinations that are not represented. Due to the nature of high-throughput data sets, there is pervasive identification of both false negatives and false positives. Similar to the underlying studies, arbitrary thresholds have been used for log2(fold change) and adj. *p* values to identify differentially regulated genes. Lower or higher thresholds will lead to the inclusion or exclusion of predicted targets, respectively.

### Gene up- and down-regulation by p53 are distinctly affected during evolution

Comparing the p53 GRN from mice to humans reveals that p53-dependent gene down-regulation is largely sustained, while gene up-regulation displays extensive variation (Fig. 2b, c). This difference stems from the two major mechanisms employed by p53 to regulate gene expression: up-regulation through direct p53 target gene binding and indirect down-regulation through the p53-p21-DREAM pathway. It has been reported that some groups of genes and regulatory elements have undergone more rapid evolution than others [10]. Here it is revealed that the p53 signaling pathway clearly contains both: more rapid evolution of directly up-regulated genes and more constrained evolution of indirectly down-regulated genes.

### Multiple mechanisms underlie conservation and divergence of the p53 GRN

The p53 GRN comprises common and species-specific target genes between mice and humans. The meta-analysis reveals that evolutionary turnover in p53REs leads to extensive variation in p53 binding profiles that ultimately result in species-specific direct p53 target genes (Figs. 3 and 4). For other TFs it has been reported that on average ~44% of TF to target gene associations are conserved between mice and humans [11]. Analysis of the one-to-one ortholog gene pairs revealed that <21% of direct p53 target genes are conserved (Fig. 5). Thus, a plethora of species-specific direct p53 target genes has been shaped through evolutionary gains and losses of p53REs.

One group of genes displays the most marked changes as it is down-regulated in one but up-regulated by p53 in the other species. This exceptional group of genes includes cell cycle genes, such as the ortholog gene pairs *PSRC1*/*Psrc1* [39, 40], *DDIAS*/*Ddias*, *GTSE1*/*Gtse1* and *E2F7*/*E2f7*. These genes have been identified as direct targets of the DREAM complex [4], which mediates cell cycle-dependent expression and p53-dependent down-regulation. One ortholog out of each of these gene pairs, however, is also directly bound and up-regulated by p53 (Fig. 2d–f, Supplementary Figures S6, S7 and S12). Consistent with previous findings regarding the regulation of *AEN* [4], these data support the notion that p53 binding can oppose DREAM-mediated down-regulation, which ultimately leads to up-regulation of direct p53 target genes despite the presence of the repressive DREAM complex. Thus, evolutionary turnover of p53REs at cell cycle genes can convert p53-dependent down-regulation into up-regulation and vice versa.

Conservation of p53's functional repertoire may be greater than indicated by direct p53 target gene conservation alone. For example, POLH and POLK both belong to the Y-family of translesion DNA synthesis polymerases and can incorporate a base opposite bulky types of DNA damage that are produced by carcinogens to allow for DNA replication despite damaged sites [53]. It has been shown in human cells that p53-dependent up-regulation of POLH results in improved bypass of DNA damage and enhanced cell survival [54]. Interestingly, human *POLH* but not mouse *Polh* and mouse *Polk* but not human *POLK* are directly up-regulated by p53 [38, 44] (Supplementary Table S6). These findings indicate that in some cases distinct direct p53 target genes may have evolved that serve a similar function in mice and humans, such as regulating DNA replication in response to DNA damage.

It remains unclear why p53 binding sites undergo more rapid evolution than binding sites of most other TFs, such as the DREAM complex. A possible explanation may be the different sizes of the DNA recognition motifs. p53REs are exceptionally large and typically contain two decameric half sites (RRRCWWGYYY). Binding sites of the p53-related TF p63 display also extensive evolutionary variation [55]. In contrast to p53REs, E2F and CHR promoter elements conferring cell cycle-dependent gene expression and DREAM binding have been described as being 7 (E2F site: TTSSSSS) or 6 (CHR: TTYGAA) nucleotides long [5, 56, 57]. It has been shown that evolution unlikely favors TF binding sites that exceed 10 bp [58, 59]. Thus, the long p53 response elements may be particularly prone to alterations compared to the short binding sites of most other TFs.

### Most direct p53 target genes are species-specific and dispensable during evolution

Previously, only a few genes have been known to differ in their p53 response between mice and humans. Here, the comprehensive analysis identified 1010 protein-coding genes that appear to differ in their p53-dependent transcriptional regulation (Supplementary Table S7). Given that the mouse-specific and the human-specific p53 target genes are not enriched for known tumor suppressor functions in the p53 response (Fig. 5) together with the lack of evolutionary conservation of their regulation, one can speculate that the rapid changes in p53REs during evolution randomly produce a plethora of species-specific direct p53 target genes that are dispensable for the function of p53. Consistent with this notion, it has been reported that trans-activation-mutants of p53 that could up-regulate only subsets of direct p53 target genes were sufficient to suppress tumor development in mice [23]. Thus, most direct p53 target genes in any species may be dispensable for the tumor suppressor function of p53.

### The small core set of common direct p53 target genes is enriched for function in apoptosis

The 86 genes that have been identified as common direct p53 targets in mice and humans (Table 1) are enriched for the p53 tumor suppressor function apoptosis (Fig. 5). The strong conservation of common p53 binding sites across vertebrates (Fig. 4c, d) indicates that this small core set of p53 target genes may be conserved through larger parts of vertebrate evolution and not only between mice and humans. This is consistent with data showing that regulating apoptosis is the main ancestral function of p53 family members [52]. Notably, mouse strains expressing cooperativity mutant p53 alleles that fail to up-regulate a larger number of pro-apoptotic p53 target genes are prone to develop numerous tumors [60]. Thus, the 86 common p53 target genes or perhaps an even smaller subset of this group may be sustained through evolution to mediate key functions of p53. Notably, about half of these genes and their encoding proteins have not been studied for their function in the p53 response [1]. Given that these genes may constitute targets that are particularly important for the function of p53, they may be interesting targets for cancer therapy.

## Materials and methods

Assessing the p53-dependent regulation of any specific gene of interest can be difficult since due to the nature of genome-wide data sets the overlap between any two data sets is often small even when the underlying data were derived from the same cell line undergoing identical treatment. The recently developed step-wise meta-analysis approach enables integration of multiple data sets to complement incomplete information in individual studies with data from other studies with noise lowered using stringent thresholds [4]. It uses a scoring system that is based on the number of data sets that agree on a gene’s regulation or on transcription factor binding sites and that can be used as a measure of confidence providing ranked maps of regulated genes.

### Meta-analysis approach

Integration and comparison of multiple data sets has been performed as described previously [4]. Briefly, publicly available data sets on p53-dependent gene regulation were curated. In most cases microarray data were available at a pre-processed stage at Gene Expression Omnibus (GEO) [61]. In these cases GEO2R was used to obtain fold expression changes and *p* values, which were adjusted for multiple testing using Benjamini Hochberg correction. For the remaining microarray data sets and for the RNA sequencing (RNA-seq) data sets fully pre-analyzed data presenting genes with their fold expression changes and adjusted (adj.) *p* values were made available by the respective authors. Common thresholds for absolute log_2_(fold-change expression) ≥0.5 and adj. *p* value ≤ 0.05 were employed to identify significantly differentially expressed genes. In some cases deviating thresholds were used to conform to settings used in the original study. Genes were ranked by *p53 Expression Score* reflecting the number of data sets that find the gene to be significantly up-regulated minus the number of data sets that find the gene to be down-regulated when p53 is active. Each expression profiling data set was tested against the sum of the remaining data sets and when more data sets agree on a gene being significantly regulated the more likely it is also identified by the remaining data set (Supplementary Figures S1 and S2). Thus, the number of data sets that agree on a gene’s regulation reflects a confidence score that this gene is regulated by p53 irrespective of treatment and cell type.

ChIP peak data sets were publicly available [62] and checked for their ability to identify known binding sites (Supplementary Figures S3–5). Intersections of binding peaks and promoter regions were calculated using ‘BETA-minus’ in ‘Cistrome’ [63, 64]. Protein binding was required to occur within 1000 and 5000 bp around the TSS for E2f4 and p53, respectively. Similar to expression profiling data sets, genes were ranked by the number of ChIP data sets that identify a binding peak near the gene’s TSS.

### Cell culture and drug treatment

C2C12 and Hepa1-6 cells (DSMZ, Braunschweig, Germany) and BJ-hTERT cells (ATCC, Manassas, Virginia, USA) were grown in Dulbecco’s modified Eagle’s medium (Thermo Fisher Scientific, Darmstadt, Germany) supplemented with 10% fetal bovine serum (FBS) (Sigma, Taufkirchen, Germany) and penicillin/streptomycin and maintained at 37 °C and 10% CO_2_. Cells were treated with dimethyl sulfoxide (DMSO; 0.15 %), Nutlin-3a (10 µM; Sigma) or 5-FU (25 µg/ml; Sigma) for 24 h.

### Chromatin immunoprecipitation, RNA extraction, reverse transcription and semi-quantitative real-time PCR

ChIP was performed with the Pierce Magnetic ChIP Kit (Thermo Fisher Scientific) following the manufacturer instructions. A total of 3 µg of p53 antibody (Cell Signaling Technology; #2524) were used per IP.

Total cellular RNA was extracted using the RNeasy Kit (Qiagen, Hilden, Germany) following the manufacturer’s protocol. One-step reverse transcription and real-time PCR were performed with a Quantstudio 5 (Thermo Fisher Scientific) using QuantiTect SYBR Green PCR Kit (Qiagen) as described previously [35].

Primer sequences are listed in Supplementary Table S2.

## Supplementary information

Supplementary Figures S1-15

Supplementary Methods

Supplementary Legends

Supplementary Table S1

Supplementary Table S2

Supplementary Table S3

Supplementary Table S4

Supplementary Table S5

Supplementary Table S6

Supplementary Table S7

Supplementary Table S8
