# Supplementary material for: Conservation and divergence of the p53 gene regulatory network between mice and humans
Source: Oncogene. 2019 Feb 1;38(21):4095–109. doi: 10.1038/s41388-019-0706-9 (PMC6755996; doi:10.1038/s41388-019-0706-9)
Supplement: Supplementary file 1 — Supplementary Figures S1-15 [file 41388_2019_706_MOESM1_ESM.pdf]

Fischer, Supplementary Figure S1

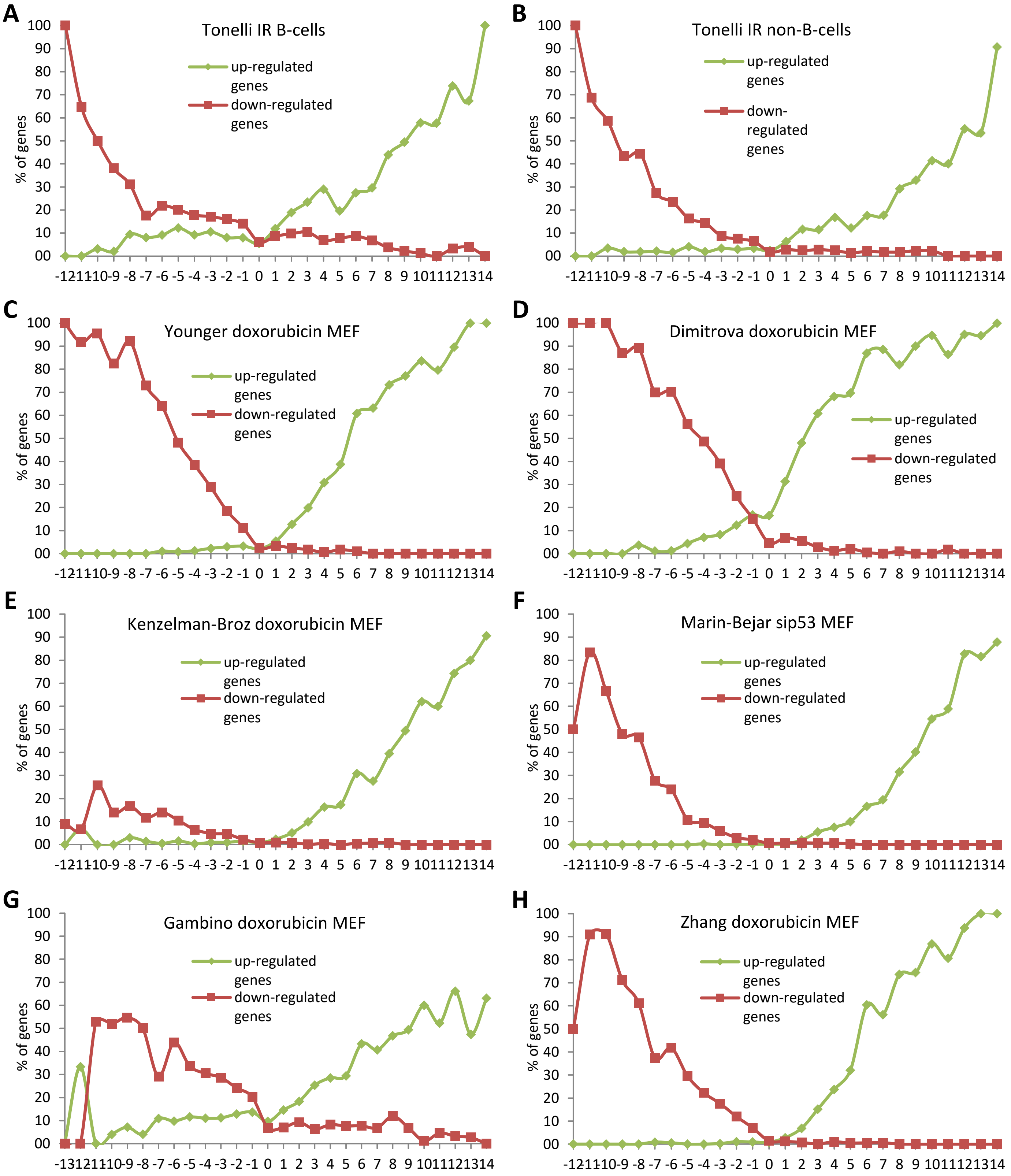

Supplementary Figure S1. Meta-analysis of p53-dependent gene expression leads to a robust combined pan cell type and treatment dataset. In each dataset on p53-dependent gene regulation a gene can be found as upregulated "+1", downregulated "-1", or not regulated "0". The number of genes identified in **(A and B)** Tonelli et al., **(C)** Younger et al., **(D)** Dimitrova et al., **(E)** Kenzelman-Broz et al., **(F)** Marin-Bejar et al., **(G)** Gambino et al., and **(H)** Zhang et al. datasets as either up-regulated or down-regulated by p53 is compared to the sum of the remaining 14 datasets.

Fischer, Supplementary Figure S2

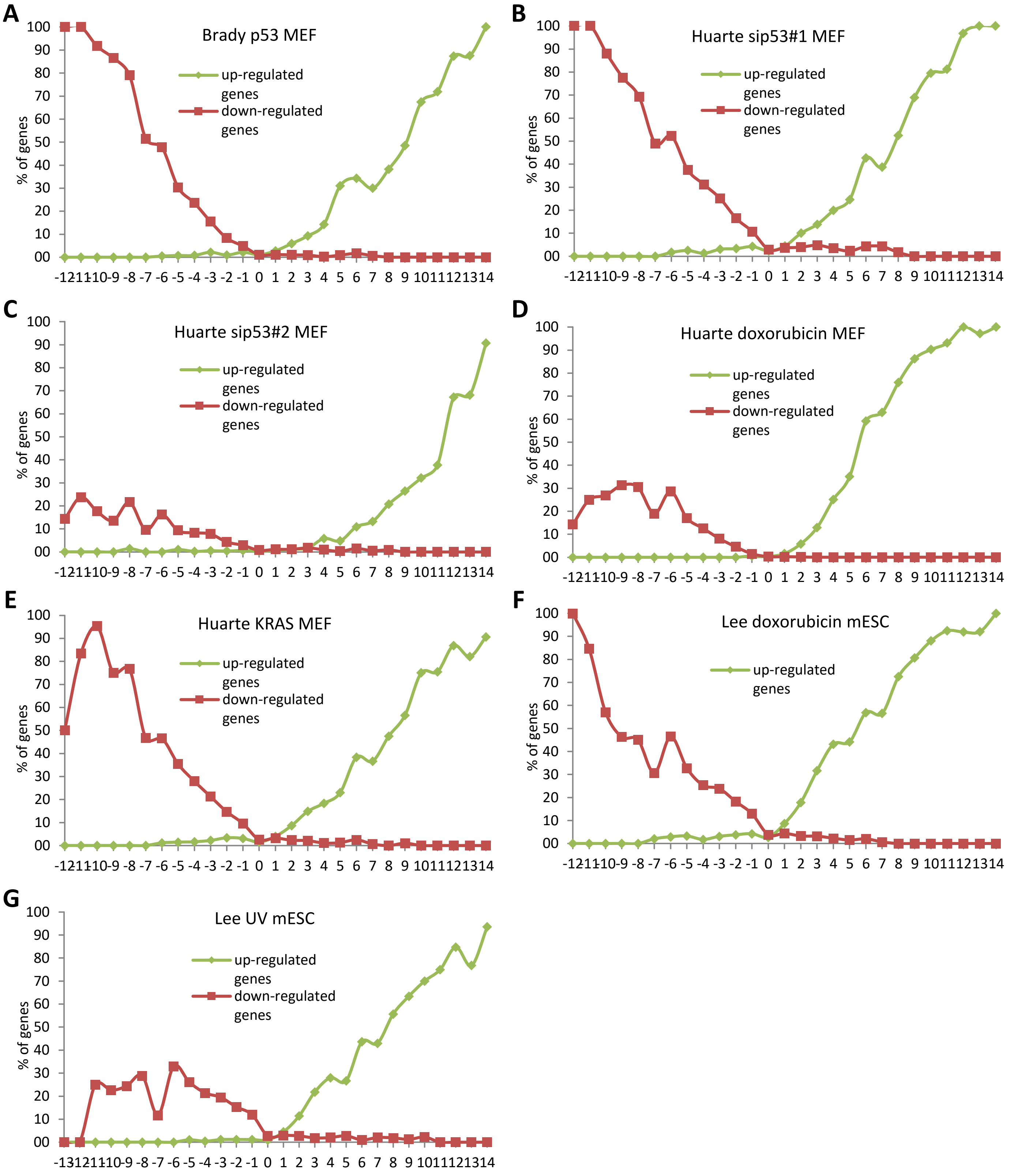

Supplementary Figure S2. Meta-analysis of p53-dependent gene expression leads to a robust combined pan cell type and treatment dataset. In each dataset on p53-dependent gene regulation a gene can be found as upregulated “+1”, downregulated “-1”, or not regulated “0”. The number of genes identified in **(A)** Brady et al., **(B - E)** Huarte et al., and **(F and G)** Lee et al. datasets as either up-regulated or down-regulated by p53 is compared to the sum of the remaining 14 datasets.

Fischer, Supplementary Figure S3

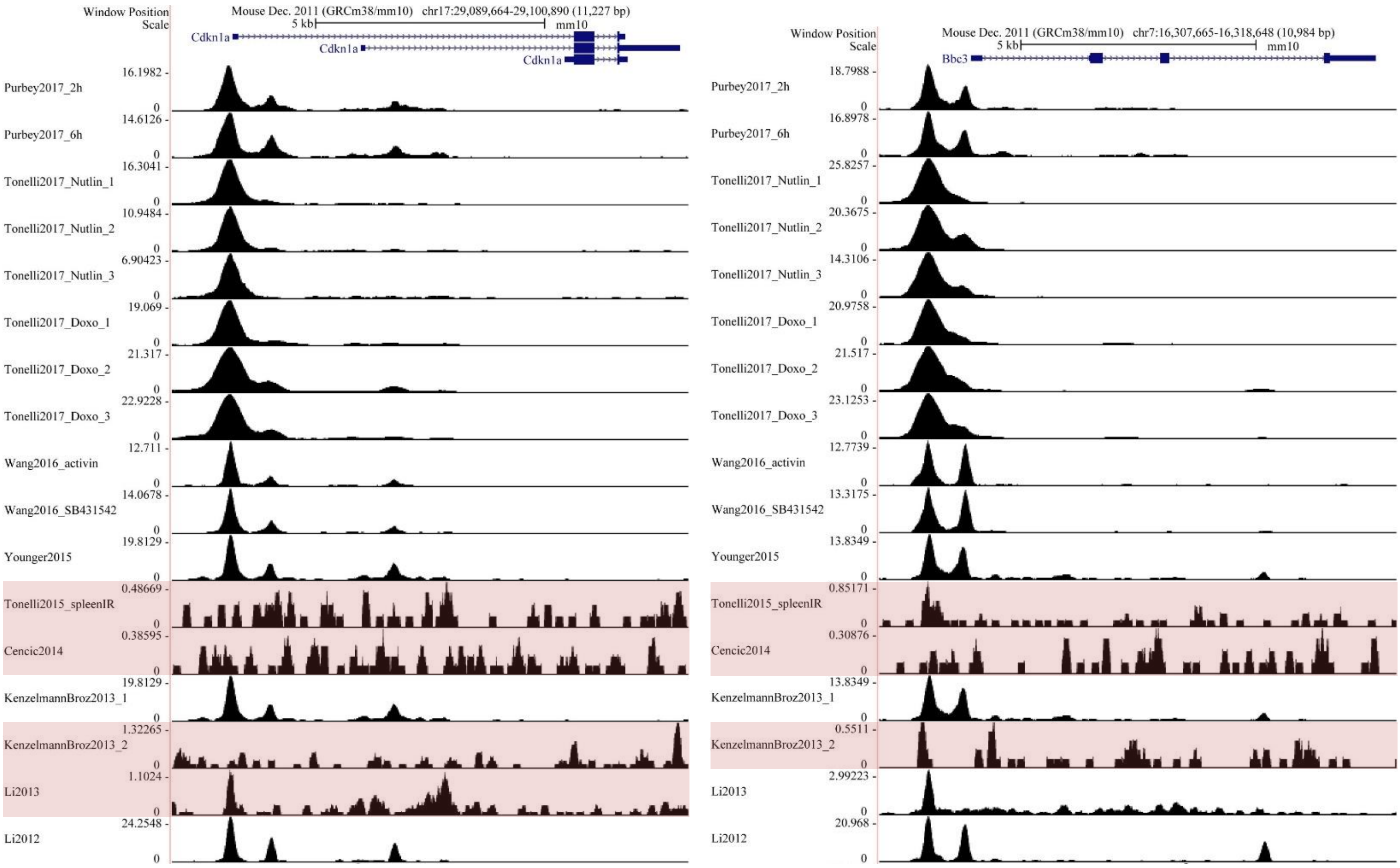

Supplementary Figure S3. UCSC genome browser tracks displaying *Cdkn1a* (left) and *Bbc3* (right). Custom tracks display the mouse p53 ChIP-Seq data. Red marked are data sets that failed to correctly identify known p53 binding sites in *Cdkn1a* or *Bbc3*.

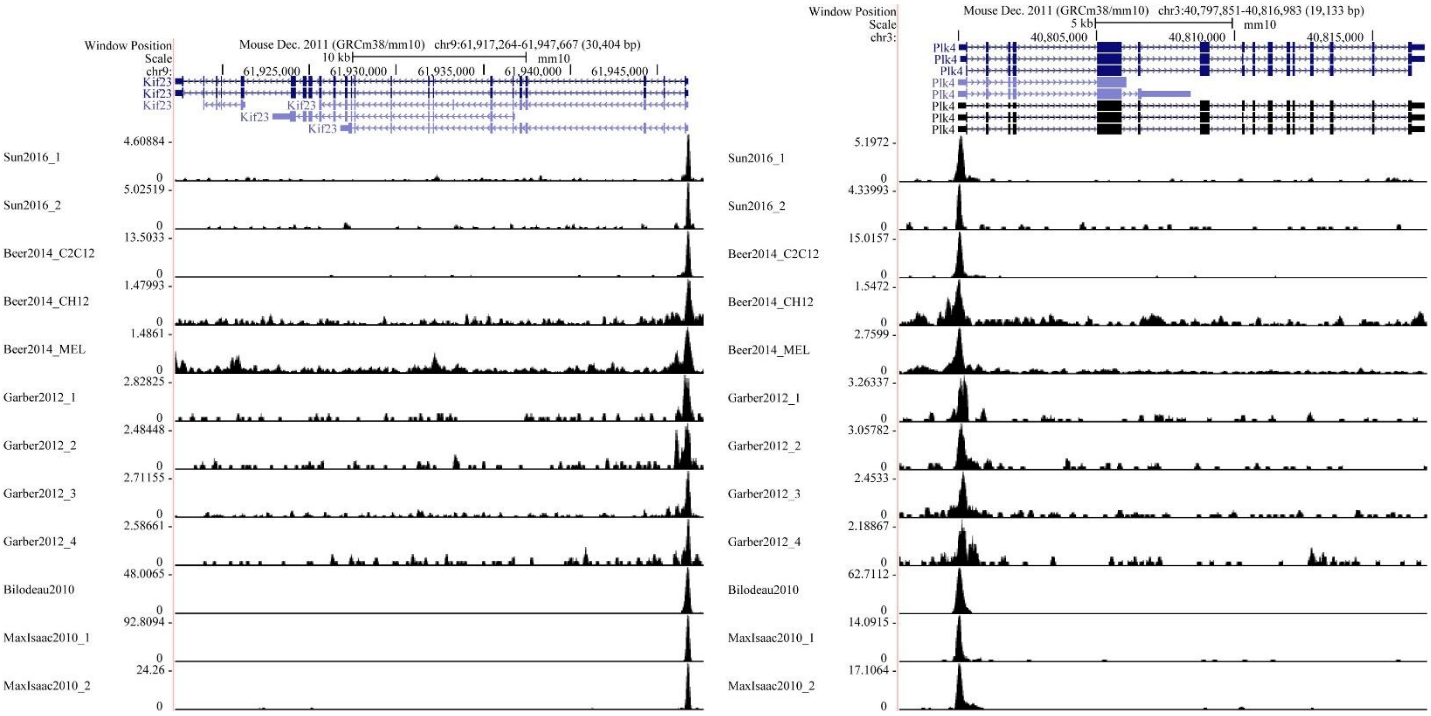

Supplementary Figure S4. UCSC genome browser tracks displaying *Kif23* (left) and *Plk4* (right). Custom tracks display the mouse E2f4 ChIP-Seq data. All data sets correctly identified known E2f4 binding sites.

Fischer, Supplementary Figure S5

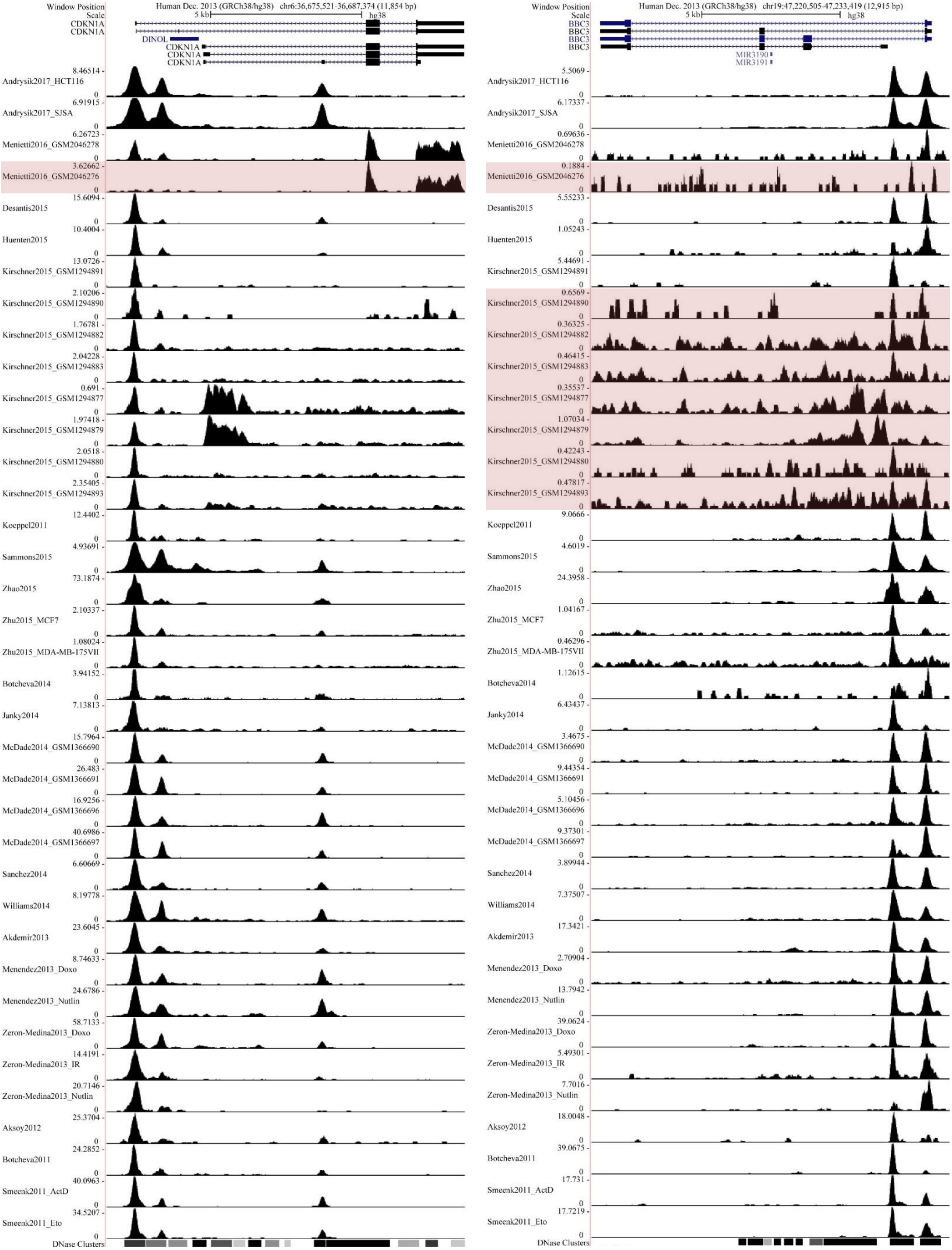

Supplementary Figure S5. UCSC genome browser tracks displaying *CDKN1A* (left) and *BBC3* (right). Custom tracks display the human p53 ChIP-Seq data. Red marked are data sets that failed to correctly identify known p53 binding sites in *CDKN1A* or *BBC3*.

Fischer, Supplementary Figure S6

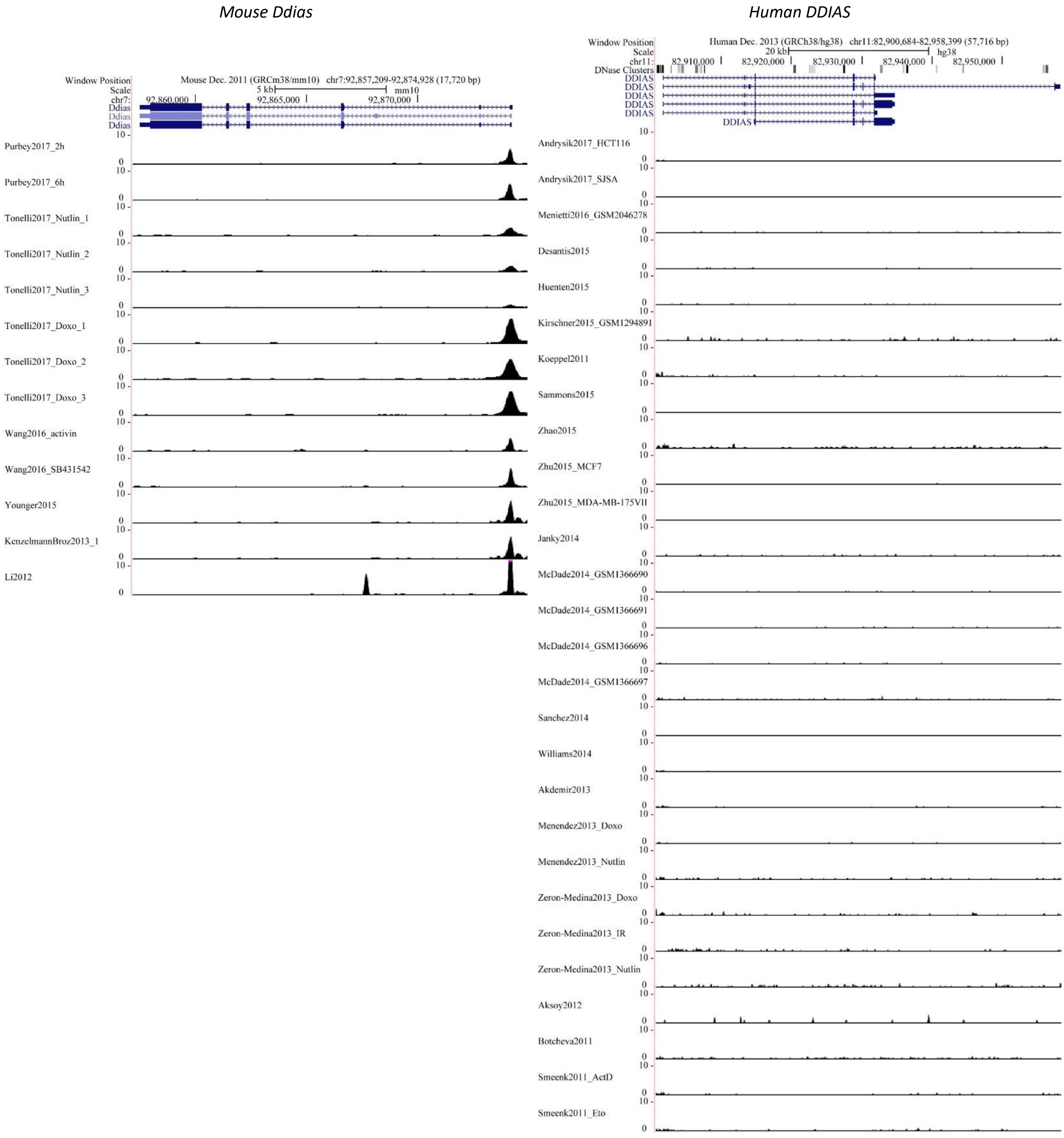

Supplementary Figure S6. UCSC genome browser tracks displaying mouse *Ddias* (left) and human *DDIAS* (right). Custom tracks display the mouse and human p53 ChIP-Seq data.

Fischer, Supplementary Figure S7

Mouse Gtse1 *Human GTSE1*

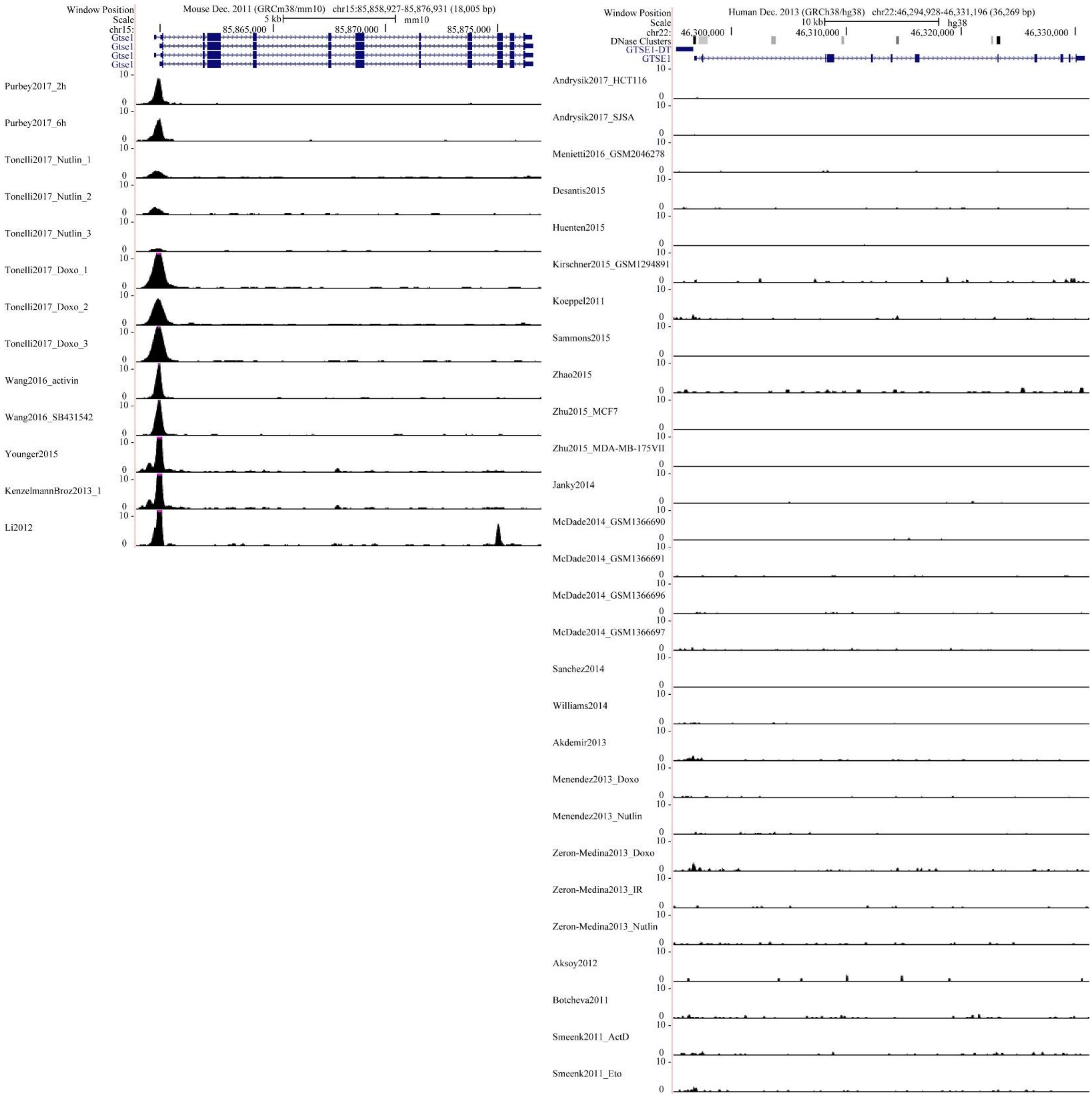

Supplementary Figure S7. UCSC genome browser tracks displaying mouse *Gtse1* (left) and human *GTSE1* (right). Custom tracks display the mouse and human p53 ChIP-Seq data.

Fischer, Supplementary Figure S8

Mouse Lpin1

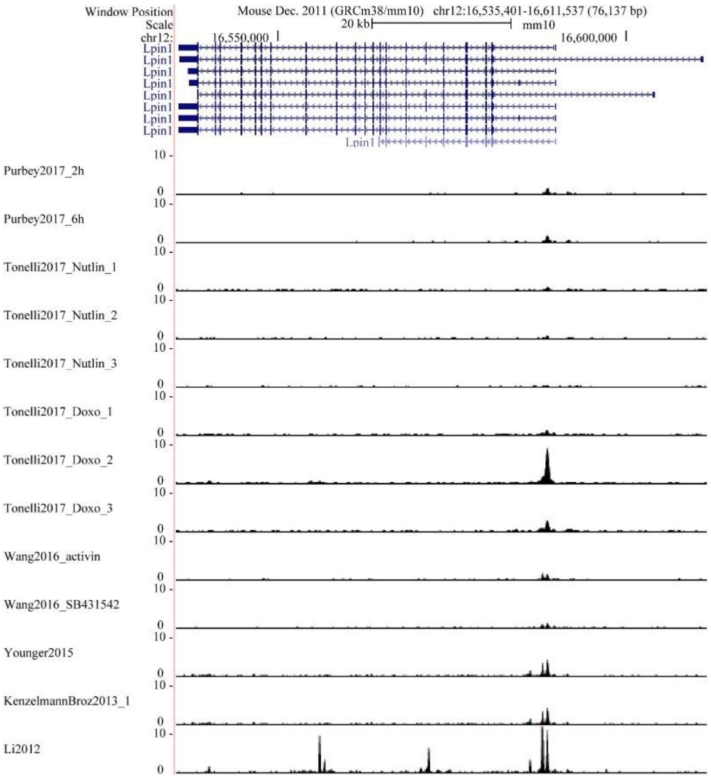

Human LPIN1

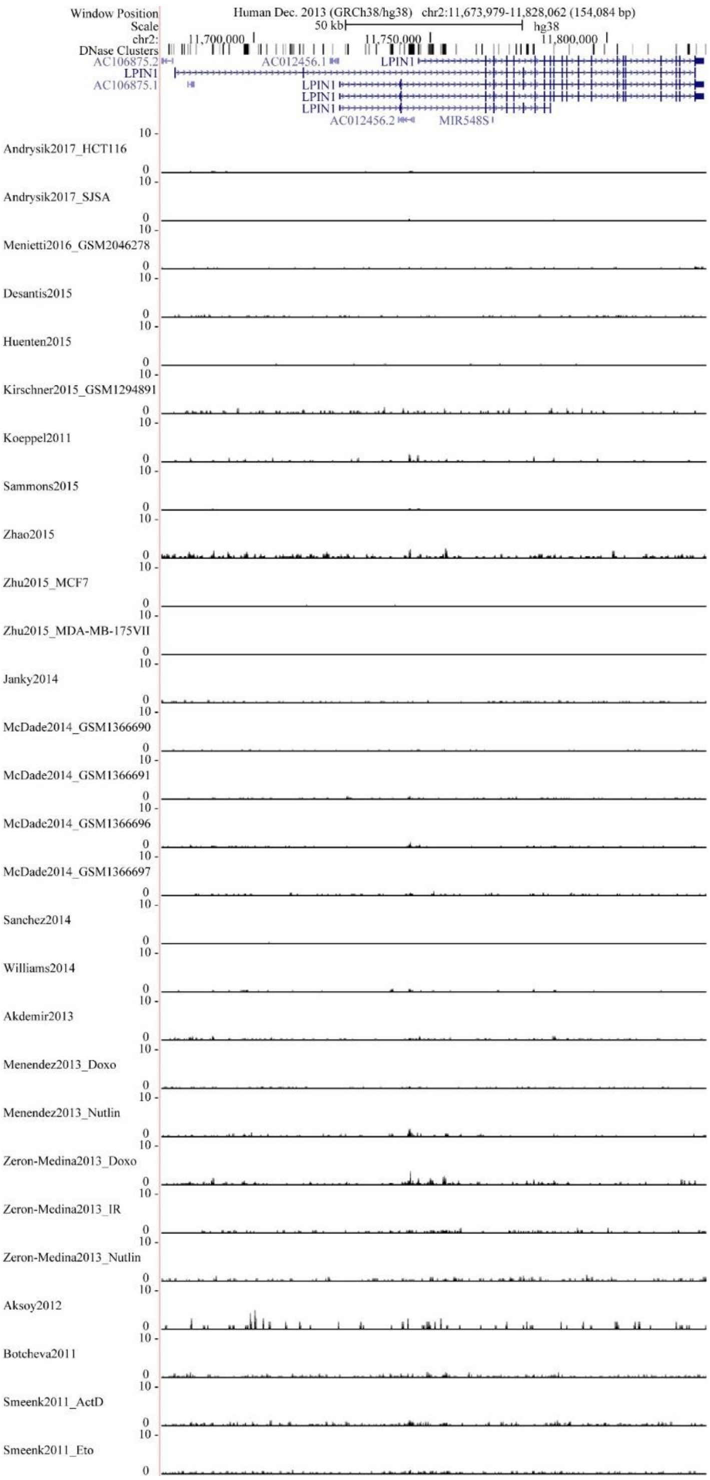

Supplementary Figure S8. UCSC genome browser tracks displaying mouse *Lpin1* (left) and human *LPIN1* (right). Custom tracks display the mouse and human p53 ChIP-Seq data.

Fischer, Supplementary Figure S9

Mouse Cpt1c

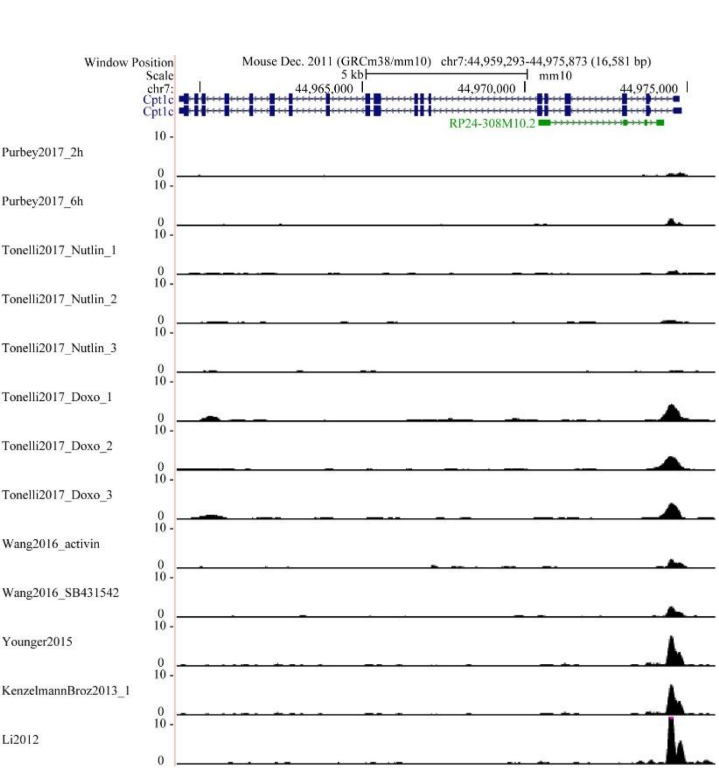

Human CPT1C

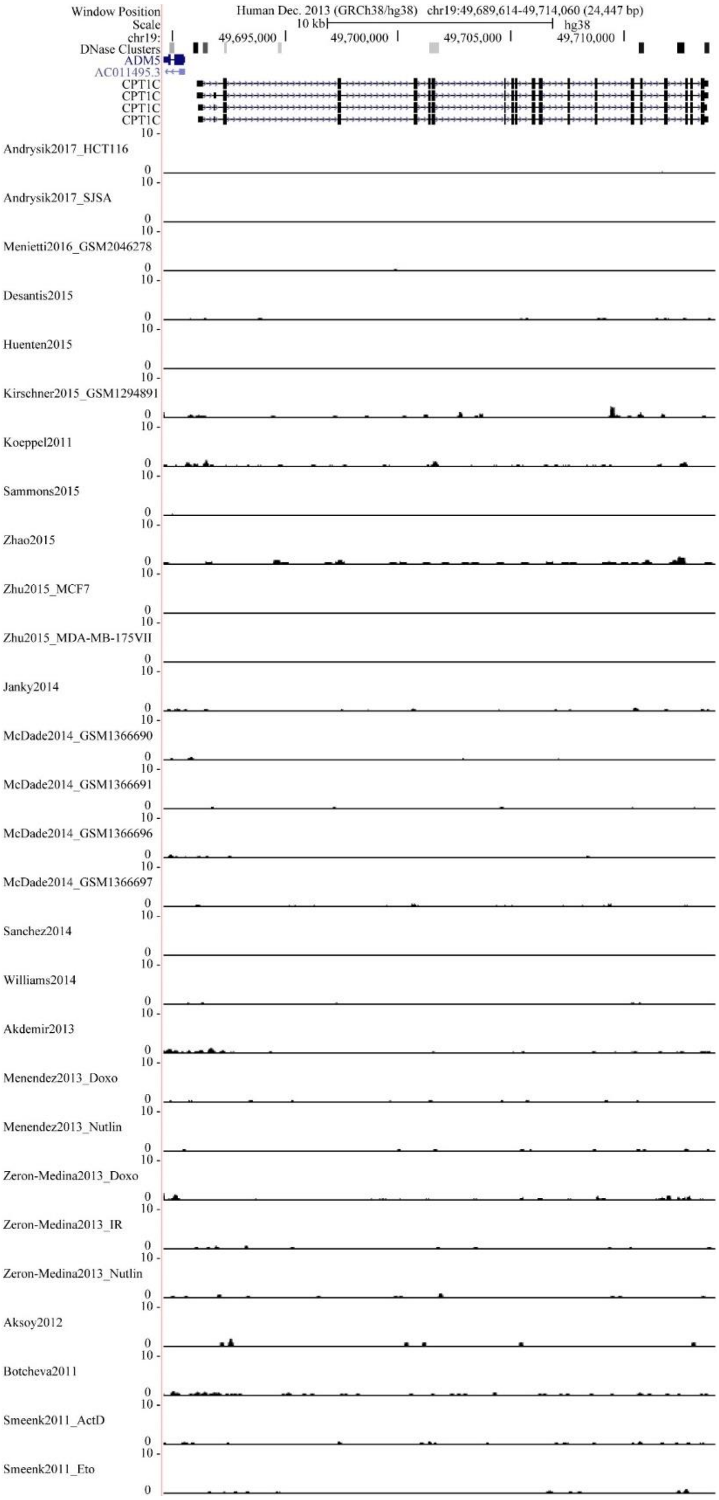

Supplementary Figure S9. UCSC genome browser tracks displaying mouse *Cpt1c* (left) and human *CPT1C* (right). Custom tracks display the mouse and human p53 ChIP-Seq data.

Fischer, Supplementary Figure S10

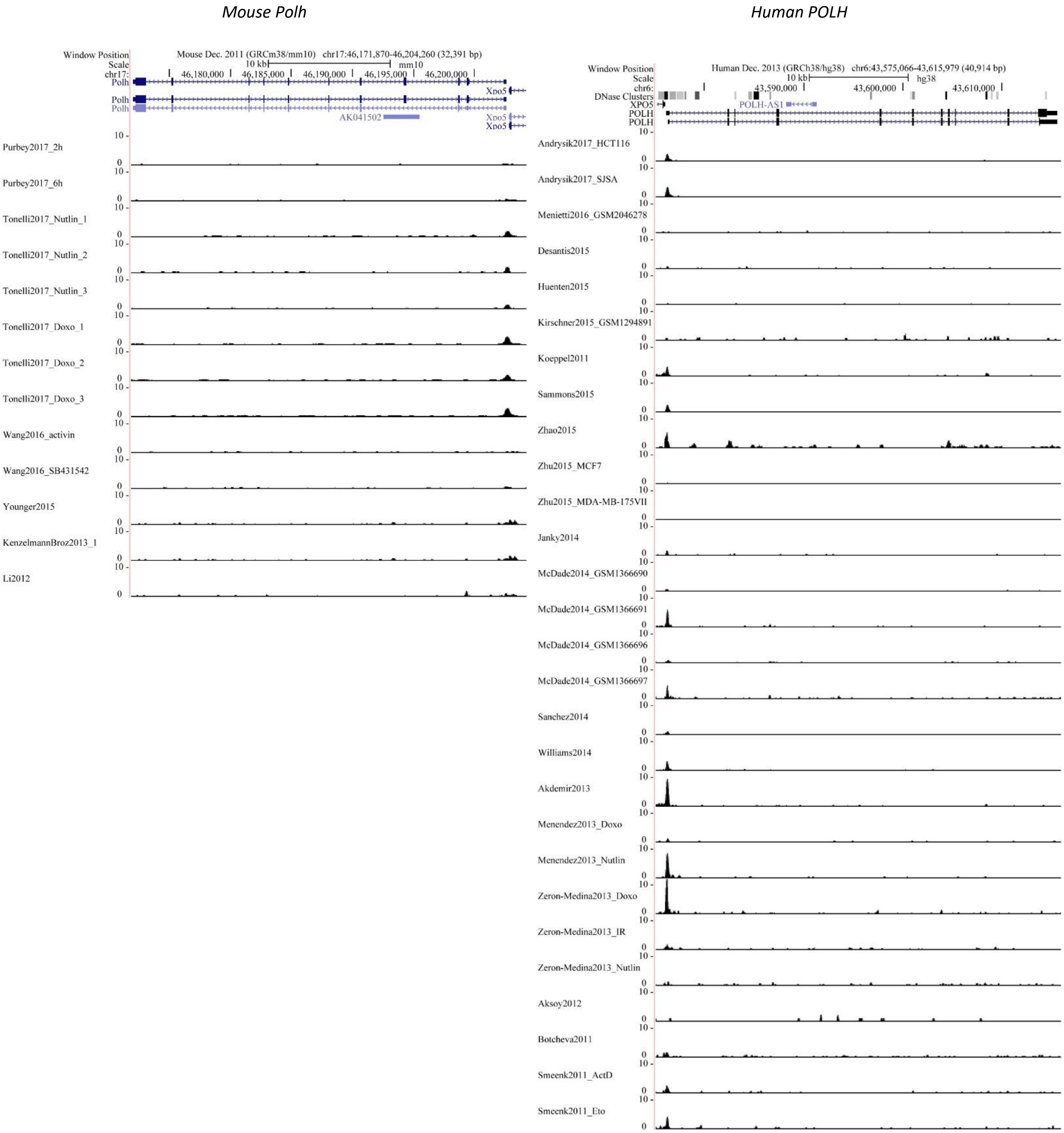

Supplementary Figure S10. UCSC genome browser tracks displaying mouse *Polh* (left) and human *POLH* (right). Custom tracks display the mouse and human p53 ChIP-Seq data.

Fischer, Supplementary Figure S11

Mouse Pank1 *Human PANK1*

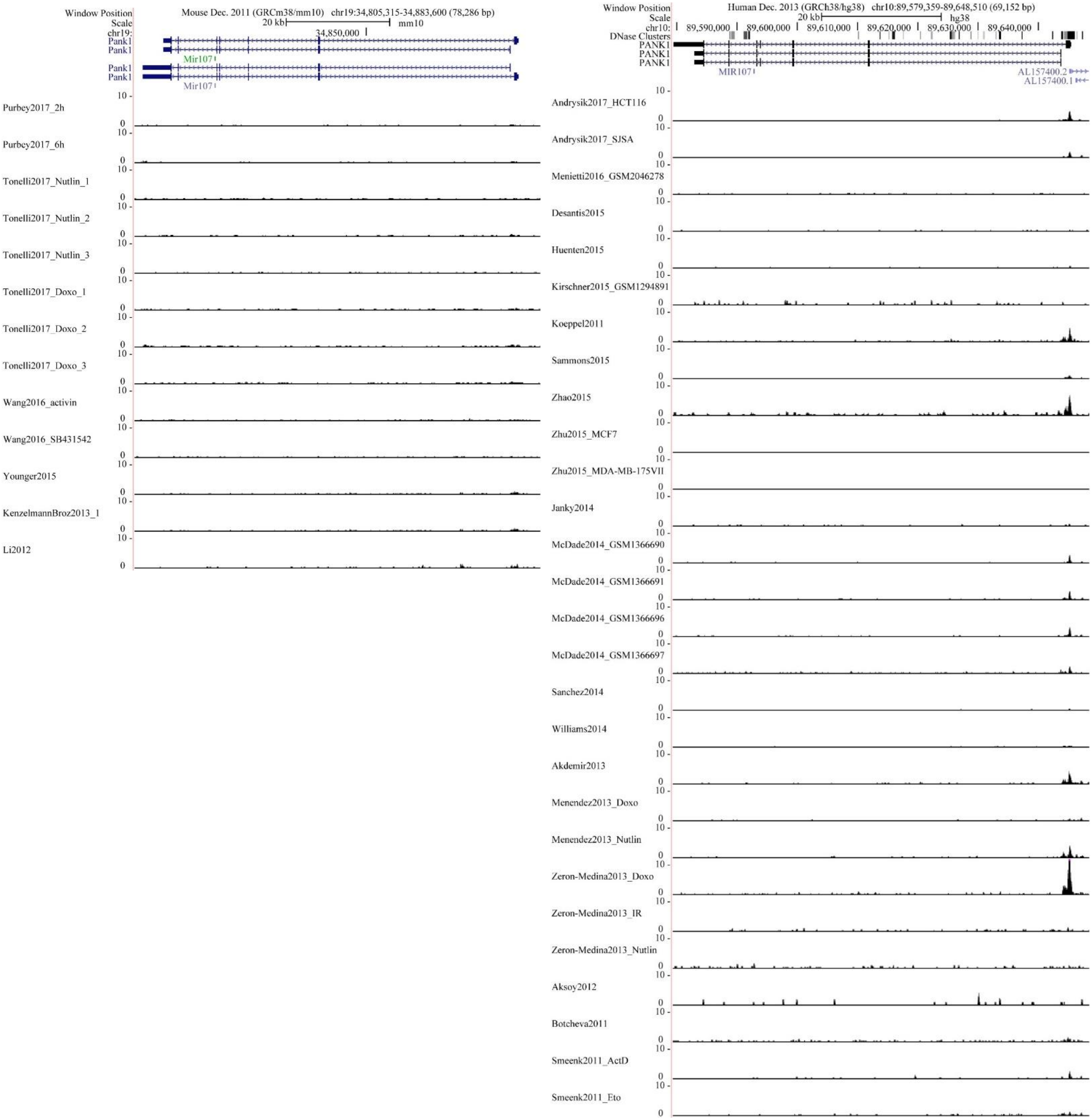

Supplementary Figure S11. UCSC genome browser tracks displaying mouse *Pank1* (left) and human *PANK1* (right). Custom tracks display the mouse and human p53 ChIP-Seq data.

Fischer, Supplementary Figure S12

Mouse E2f7 *Human E2F7*

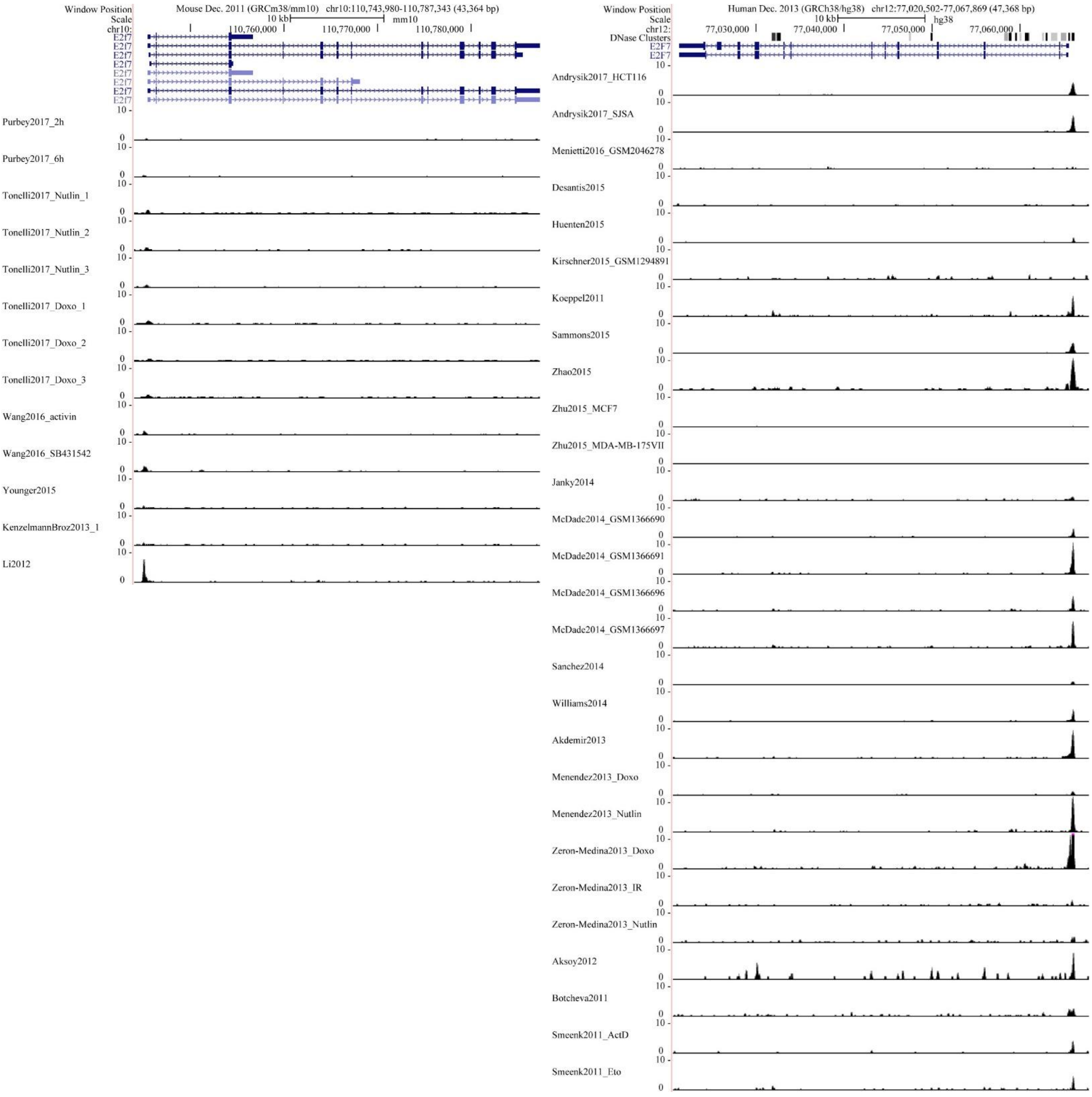

Supplementary Figure S12. UCSC genome browser tracks displaying mouse *E2f7* (left) and human *E2F7* (right). Custom tracks display the mouse and human p53 ChIP-Seq data.

Fischer, Supplementary Figure S13

Mouse Tigar *Human TIGAR*

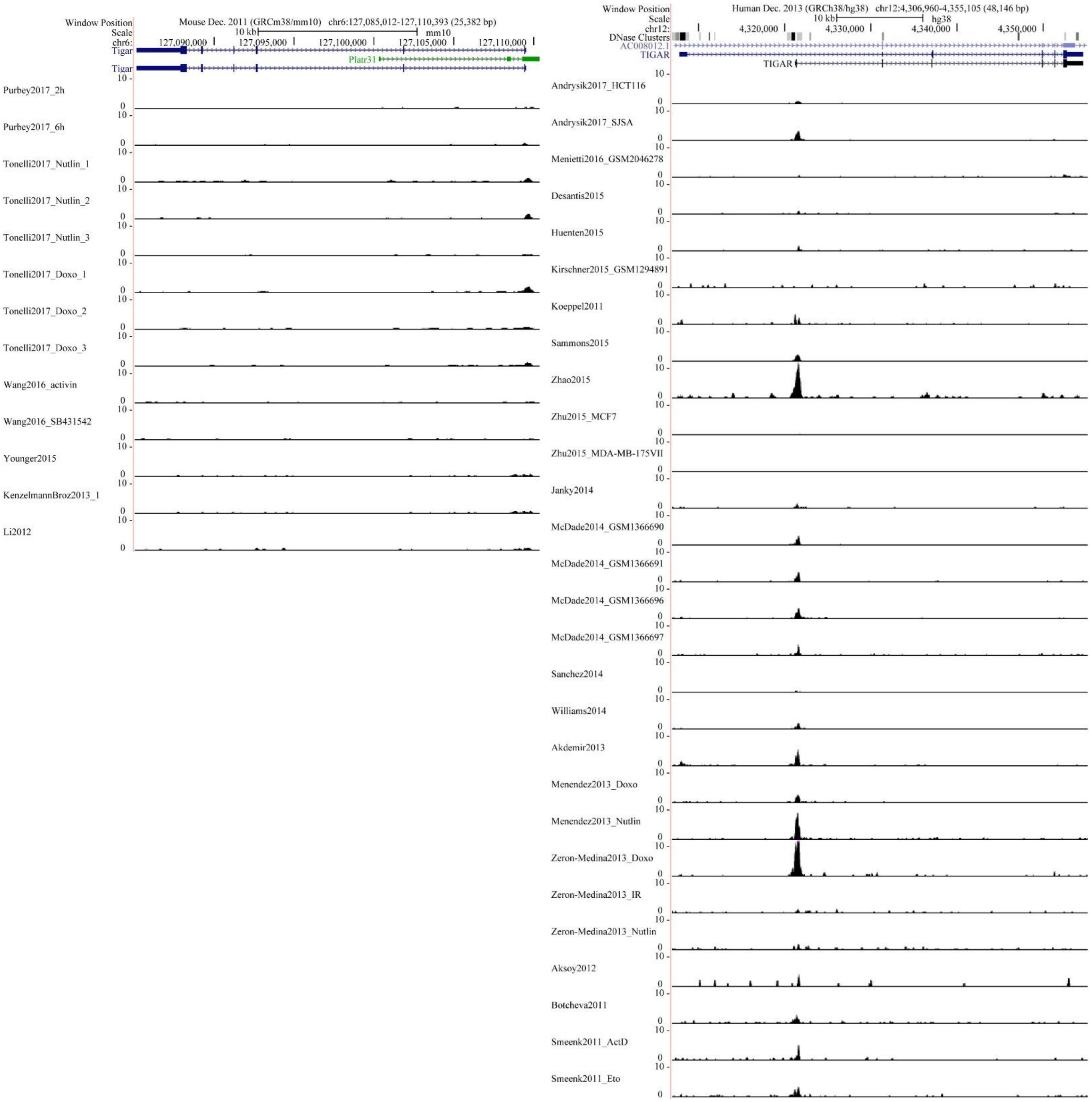

Supplementary Figure S13. UCSC genome browser tracks displaying mouse *Tigar* (left) and human *TIGAR* (right). Custom tracks display the mouse and human p53 ChIP-Seq data.

Fischer, Supplementary Figure S14

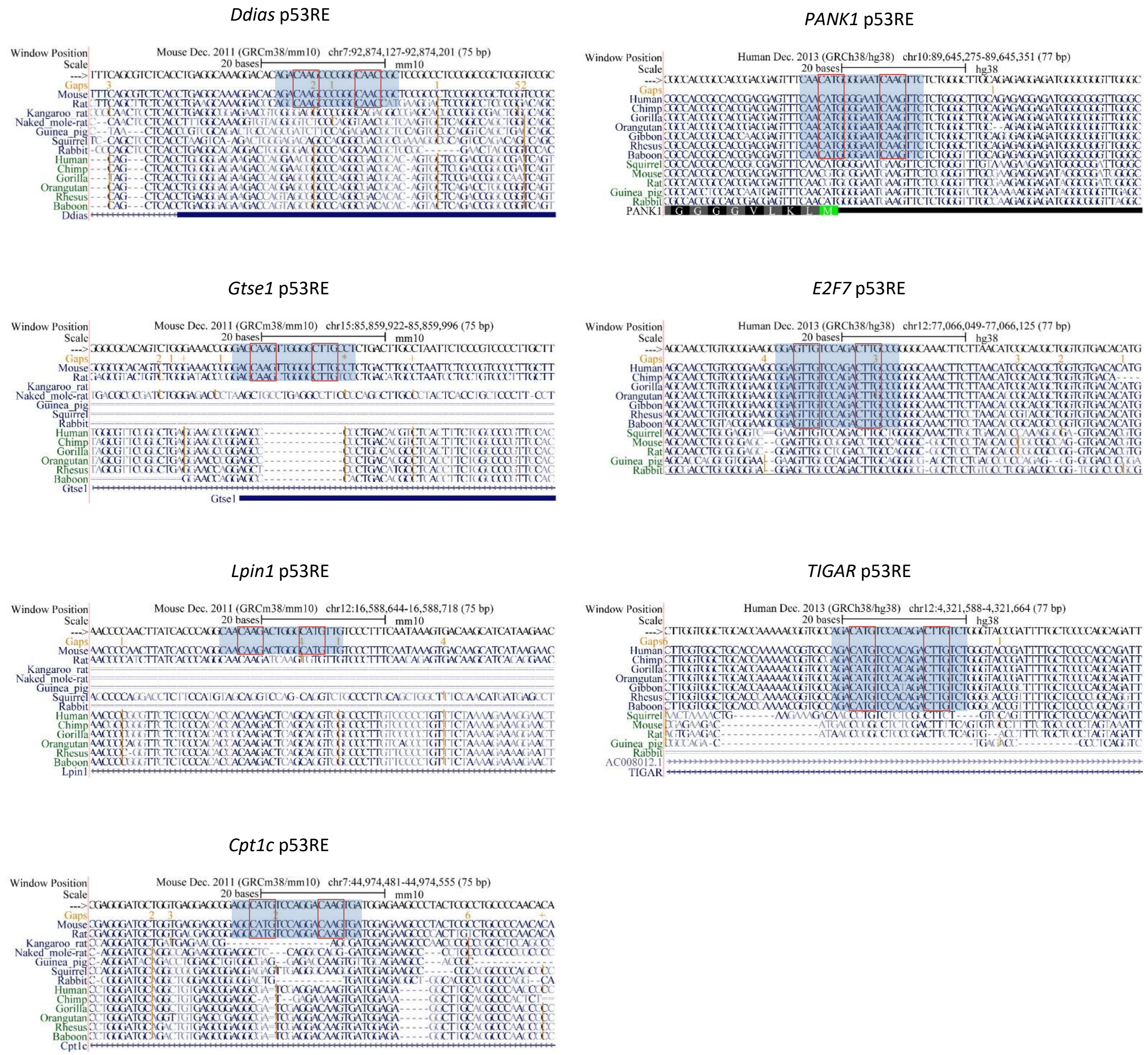

Supplementary Figure S14. UCSC genome browser alignment tracks from primates, rodents and rabbit focused on p53REs that are bound by p53 in mouse *Ddias*, *Gtse1*, *Lpin1* and *Cpt1c* and human *PANK1*, *E2F7* and *TIGAR*.

Fischer, Supplementary Figure S15

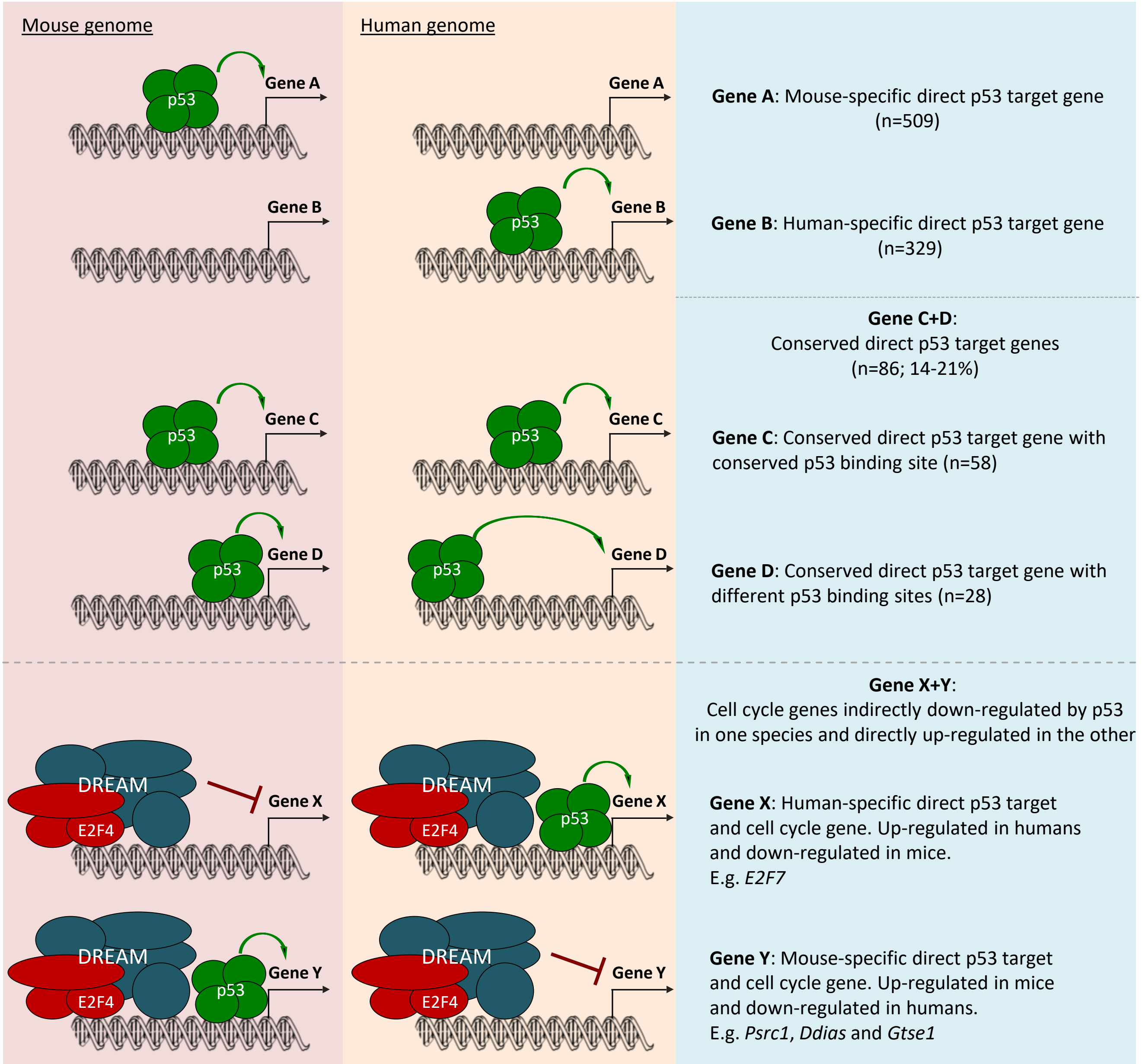

Supplementary Figure S15. Categories of direct p53 target genes. Direct p53 target genes were identified as conserved or as mouse- or human-specific. Conserved direct target genes were grouped into containing an overlapping p53 binding site or p53 binding sites that differ in their location relative to the TSS (non-overlapping). The most marked differences in p53-dependent regulation between mice and humans are displayed by cell cycle genes that are DREAM targets and up-regulated and bound by p53 in one species, but down-regulated and not bound by p53 in the other species.
