# Supplementary material for: Conservation and divergence of the p53 gene regulatory network between mice and humans
Source: Oncogene. 2019 Feb 1;38(21):4095–109. doi: 10.1038/s41388-019-0706-9 (PMC6755996; doi:10.1038/s41388-019-0706-9)
Supplement: Supplementary file 2 — Supplementary Methods [file 41388_2019_706_MOESM2_ESM.pdf]

Supplementary Methods

Meta-analysis of p53-dependent gene regulation

Gene identifiers were mapped to Ensembl Gene IDs primarily using the Ensembl annotation data [1] and when no Ensembl annotation was available annotations from the Mouse Genome Database [2] were used (NCBIM37). Ensembl annotations for the canonical transcripts for the mouse genome version mm9 was retrieved from Ensembl BioMart [1,3].

Expression data for genes was extracted from 15 datasets on p53-dependent regulation published in 10 studies [4–13]. Expression data for 20,912 genes was available from at least three datasets. For these genes the genomic location is shown (Supplementary Table S1), which is defined by chromosome, strand and gene start and end. Expression values of the analyzed genes were compiled and classified into down-regulated (−1), up-regulated (+1), and not-regulated (0) by p53. The individual datasets were curated as followed:

The pre-analyzed p53 gene expression profiling dataset of Tonelli et al. from IR treated B-cells and non-B-cells (GSE71180) was kindly provided by Claudia Tonelli and Bruno Amati [13,14], and was used with thresholds of adj.p-value ≤ 0.05 and an absolute log2(fold-change expression) ≥ 0.5.

The pre-analyzed p53 gene expression profiling dataset of Younger et al. from doxorubicin treated MEFs was kindly provided by Scott Younger [12], and was used with thresholds of q-value ≤ 0.05 and an absolute log2(fold-change expression) ≥ 0.5.

The pre-analyzed p53 gene expression profiling dataset of Dimitrova et al. from doxorubicin treated wild-type MEFs (GSM1278606-08 against GSM1278603-05; GSE52957) was kindly provided by Jesse Ray Zamudio and Tyler Jacks [11], and was used with thresholds of adj.p-value ≤ 0.05 and an absolute log2(fold-change expression) ≥ 0.5.

The expression data of Marin-Bejar et al. from siTP53 treated 3T3 MEFs was retrieved from the GEO depository GSE46247 [10]. The dataset was curated using GEO2R [15] for GSM1126988-90 against GSM1126985-87. P-values were adjusted using Benjamini Hochberg correction in GEO2R. To consider a gene as regulated, thresholds of adj.p-value value ≤ 0.05 and an absolute log2(fold-change expression) ≥ 0.5 were used.

The pre-analyzed p53 gene expression profiling dataset of Kenzelmann-Broz et al. from doxorubicin treated MEFs was retrieved from the deposited Supplementary Table S3 in Kenzelmann-Broz et al. [7], that employed a threshold of adj.p-value ≤ 0.1.

The pre-analyzed p53 gene expression profiling dataset of Zhang et al. from doxorubicin treated MEFs was retrieved from the deposited Supplementary Tables S1 and S2 in Zhang et al. [8], that employed a threshold of p-value ≤ 0.001.

The expression data of Brady et al. from p53 knock-in compared to p53 knock-out MEFs was retrieved from the GEO depository GSE27901 [6]. The dataset was curated using GEO2R for GSM688946-48 and GSM688955-57 against GSM688949-54. P-values were adjusted using Benjamini Hochberg correction in GEO2R. To consider a gene as regulated, thresholds of adj.p-value value ≤ 0.05 and an absolute log2(fold-change expression) ≥ 0.5 were used.

The expression data of Lee et al. from doxorubicin and UV treated mESCs (R1E) was retrieved from the GEO depository GSE16428 [4]. The doxorubicin dataset was curated using GEO2R for GSM412779-82 against GSM412775-78. The UV dataset was curated using GEO2R for GSM412783-86 against GSM412775-78. P-values were adjusted using Benjamini Hochberg correction in GEO2R. To consider a gene as regulated, thresholds of adj.p-value value ≤ 0.05 and an absolute log2(fold-change expression) ≥ 0.5 were used.

The expression data of Huarte et al. from siTP53, doxorubicin and KRAS treated MEFs was retrieved from the GEO depository GSE21761 [5]. One siTP53 dataset was curated using GEO2R for GSM542421, GSM542425 and GSM542429 against GSM542422, GSM542426 and GSM542430. A second siTP53 dataset was curated using GEO2R for GSM542443, GSM542446 and GSM542449 against GSM542445 and GSM542448. The doxorubicin dataset was curated using GEO2R for GSM542417 and GSM542418 against GSM542409-14. The KRAS dataset was curated using GEO2R for GSM542455, GSM542456, GSM542461 and GSM542462 against GSM542451, GSM542457 and GSM542463. P-values were adjusted using Benjamini Hochberg correction in GEO2R. To consider a gene as regulated, thresholds of adj.p-value value ≤ 0.05 and an absolute log2(fold-change expression) ≥ 0.5 were used.

Mapping of orthologous gene pairs

Human orthologs were mapped to mouse genes primarily based on Ensembl orthology data [1] and when no Ensembl orthology data was available HGNC orthology data [16] was used. 15,569 ortholog gene pairs were identified that correspond to exactly one mouse and exactly one human gene (one-to-one-orthologs) and for which both the mouse and the human ortholog was assigned a *p53 Expression Score*. One-to-many and many-to-many orthologs have been excluded from the analysis given the limitations to correctly assess the conservation of their regulation.

Gene ontology (GO) analysis

The enrichment of gene ontology (GO) terms of biological processes in a particular gene list was performed using the online tool PANTHER Version 14.0 [17]

Protein binding data

Protein binding data from ChIP-seq experiments were collected from CistromeDB [18]. As quality check, p53 binding data was required to identify known p53 binding sites in the *Cdkn1a* and *Bbc3* genes (Supplementary Figure S3 and S4), and E2f4 binding data was requited to identify known E2f4 binding sites in *Kif23* and *Plk4* (Supplementary Figure S5). Data sets that failed to meet the quality criteria were not included and are makred red in Table SM1. When replicate experiments were available, all peaks were used that have been identified in at least two replicates. Human DREAM binding data was obtained from the previous meta-analysis [19]. To map binding peaks to the mouse genome assembly mm10 or the human genome assembly hg38 UCSC liftover was used [20,21]. Intersections of binding data and promoter regions were calculated using 'BETA-minus' [22] in 'Cistrome' [23]. For target gene analysis, protein binding was required to occur within 5,000 bp around the TSS in case of p53 and 1,000 bp (consistent with previous analyses [19,24]) around the TSS in case of E2f4. The location of the peak that is closest to the TSS is displayed.

Genome-wide studies of TF binding profiles employ the chromatin immunoprecipitation (ChIP) technique, which can produce fixation artifacts [25–27].

Table SM1. Protein binding data sets obtained from CistromeDB. Data sets that did not pass quality check are marked red.

ChIP-seq studies			
p53 mouse			
Study	GEO Ids	CistromeDB ID	Number of peaks
Purbey et al., 2017	GSM2698330	86309	1494
Purbey et al., 2017	GSM2698331	86891	4232
Tonelli et al., 2017	GSM2114414	74441	2984
Tonelli et al., 2017	GSM2114415	74440	4081
Tonelli et al., 2017	GSM2114416	74439	1697
Tonelli et al., 2017	GSM2114417	74438	11676
Tonelli et al., 2017	GSM2114418	74437	9138
Tonelli et al., 2017	GSM2114419	74436	10481
Wang et al., 2016	GSM1782925	72459	3290
Wang et al., 2016	GSM1782926	72458	3261
Tonelli et al., 2015	GSM1828859	55942	160

Younger et al., 2015	GSM1342502	52169	10321
Cencic et al., 2014	GSM1385971	48776	175
Li et al., 2013	GSM1209644	34463	5869
Kenzelmann-Broz et al., 2013	GSM1126945	35105	10321
Kenzelmann-Broz et al., 2013	GSM1126947	35097	286
Li et al., 2012	GSM647225	8862	12348
E2f4 mouse			
Study	GEO Ids	CistromeDB ID	Number of peaks
Sun et al., 2016	GSM2040953	68276	1220
Sun et al., 2016	GSM2040954	68275	496
Sun et al., 2016	GSM2040956	68273	1113
Beer et al, 2014	GSM912912	46385	7790
Beer et al, 2014	GSM912914	46466	19403
Beer et al, 2014	GSM915187	34416	8992
Garber et al., 2012	GSM881060	37912	599
Garber et al., 2012	GSM881061	37892	636
Garber et al., 2012	GSM881062	37898	766
Garber et al., 2012	GSM881063	37902	654
Bilodeau et al., 2010	GSM602777	5877	9156
MacIsaac et al., 2010	GSM427091	3226	7629
MacIsaac et al., 2010	GSM427094	114	7194
p53 human			
Study	GEO Ids	CistromeDB ID	Number of peaks
Andrysik et al., 2017	GSM2296272	82544	9393
Andrysik et al., 2017	GSM2296278	82549	19220
Menietti et al., 2016	GSM2046276	69457	5632
Menietti et al., 2016	GSM2046278	69456	12393
Desantis et al., 2015	GSM1468849	53285	781
Huenten et al., 2015	GSM1638967	55387	2176
Kirschner et al., 2015	GSM1294877	52090	6690
Kirschner et al., 2015	GSM1294879	52092	22267
Kirschner et al., 2015	GSM1294880	52093	170
Kirschner et al., 2015	GSM1294882	52095	437
Kirschner et al., 2015	GSM1294883	52096	328
Kirschner et al., 2015	GSM1294890	52103	1442
Kirschner et al., 2015	GSM1294891	52104	1012
Kirschner et al., 2015	GSM1294893	52106	385
Sammons et al., 2015	GSM1418970	50361	8646
Younger et al., 2015	GSM1342488	52165	7015
Younger et al., 2015	GSM1342494	52167	6445
Zhao et al., 2015	GSM1917772	68462	1900
Zhu et al., 2015	GSM1429753	54519	907
Zhu et al., 2015	GSM1429754	54520	770
Botcheva et al., 2014	GSM1417250	50346	274
Janky et al., 2014	GSM1146168	47299	895

McDade et al., 2014	GSM1366690	44763	5645
McDade et al., 2014	GSM1366691	44764	9504
McDade et al., 2014	GSM1366696	44769	9575
McDade et al., 2014	GSM1366697	44770	6841
Sanchez et al., 2014	GSM1412744	50345	3353
Williams et al., 2014	GSM1348340	48445	8481
Akdemir et al., 2013	GSM981236	43999	9934
Menendez et al., 2013	GSM1133484	33077	2532
Menendez et al., 2013	GSM1133486	33075	22837
Zeron-Medina et al., 2013	GSM1142696	41496	20659
Zeron-Medina et al., 2013	GSM1142700	41500	1744
Zeron-Medina et al., 2013	GSM1142702	41502	640
Aksoy et al., 2012	GSM1048851	40287	2142
Botcheva et al., 2011	GSM783262	5832	571
Koeppel et al., 2011	GSM501691	6547	9674
Smeenk et al., 2011	GSM545807	2796	1893
Smeenk et al., 2011	GSM545808	2795	2878

Combining multiple data sets on protein binding

Bedtools ‘multiinter’ [28] was used to intersect 28 human p53 ChIP peak files, 9 mouse p53 peak files, 7 mouse E2f4 peak files and 9 human DREAM peak files. BedGraph files were converted to BigWig files using the UCSC tool ‘BedGraphToBigWig’ [20]. To calculate conservation scores with the tool ‘Conservation Plot’ [23], binding sites supported by only a small number of data sets were removed for further analyses (see main text). To identify overlapping and non-overlapping peaks, bedtools ‘intersect’ was employed.

Visualization

Boxplots and violin plots were generated using the online tool ‘BoxPlotR’ [29]. Conservation plots displaying the average vertebrate PhastCons score [30] were generated using the ‘Conservation Plot’ tool in ‘Cistrome’ [23].

Motif search

DNA binding motifs were identified using the ‘known motifs’ in HOMER v4.9 [31] with default options and *-size given*.

Annotation of genome features

The ‘Cis-regulatory Element Annotation System’ (CEAS) tool in ‘Cistrome’ [23] was used to identify the enrichment of binding sites at genome features.

References

1 Aken BL, Achuthan P, Akanni W, Amode MR, Bernsdorff F, Bhai J *et al.* Ensembl 2017. *Nucleic Acids Res* 2017; **45**: D635–D642.

2 Blake JA, Eppig JT, Kadin JA, Richardson JE, Smith CL, Bult CJ. Mouse Genome Database (MGD)-2017: community knowledge resource for the laboratory mouse. *Nucleic Acids Res* 2017; **45**: D723–D729.

3 Kinsella RJ, Kähäri A, Haider S, Zamora J, Proctor G, Spudich G *et al.* Ensembl BioMarts: A hub for data retrieval across taxonomic space. *Database* 2011; **2011**: bar030.

4 Lee K-H, Li M, Michalowski AM, Zhang X, Liao H, Chen L *et al.* A genomewide study identifies the Wnt signaling pathway as a major target of p53 in murine embryonic stem cells. *Proc Natl Acad Sci U S A* 2010; **107**: 69–74.

5 Huarte M, Guttman M, Feldser D, Garber M, Koziol MJ, Kenzelmann-Broz D *et al.* A large intergenic noncoding RNA induced by p53 mediates global gene repression in the p53 response. *Cell* 2010; **142**: 409–419.

6 Brady CA, Jiang D, Mello SS, Johnson TM, Jarvis LA, Kozak MM *et al.* Distinct p53 transcriptional programs dictate acute DNA-damage responses and tumor suppression. *Cell* 2011; **145**: 571–583.

7 Kenzelmann Broz D, Mello SS, Bieging KT, Jiang D, Dusek RL, Brady CA *et al.* Global genomic profiling reveals an extensive p53-regulated autophagy program contributing to key p53 responses. *Genes Dev* 2013; **27**: 1016–1031.

8 Zhang X, He Y, Lee KH, Dubois W, Li Z, Wu X *et al.* Rap2b, a novel p53 target, regulates p53-mediated pro-survival function. *Cell Cycle* 2013; **12**: 1279–1291.

9 Gambino V, De Michele G, Venezia O, Migliaccio P, Dall'Olio V, Bernard L *et al.* Oxidative stress activates a specific p53 transcriptional response that regulates cellular senescence and aging. *Aging Cell* 2013; **12**: 435–445.

10 Marín-Béjar O, Marchese FP, Athie A, Sánchez Y, González J, Segura V *et al.* Pint lincRNA connects the p53 pathway with epigenetic silencing by the Polycomb repressive complex 2. *Genome Biol* 2013; **14**: R104.

11 Dimitrova N, Zamudio JR, Jong RM, Soukup D, Resnick R, Sarma K *et al.* LincRNA-p21 Activates p21 In cis to Promote Polycomb Target Gene Expression and to Enforce the G1/S Checkpoint. *Mol Cell* 2014; **54**: 777–790.

12 Younger ST, Kenzelmann-Broz D, Jung H, Attardi LD, Rinn JL. Integrative genomic analysis reveals widespread enhancer regulation by p53 in response to DNA damage. *Nucleic Acids Res* 2015; **43**: 4447–4462.

13 Tonelli C, Morelli MJ, Bianchi S, Rotta L, Capra T, Sabò A *et al.* Genome-wide analysis of p53 transcriptional programs in B cells upon exposure to genotoxic stress in vivo. Oncotarget. 2015; **6**: 24611–26.

14 Tonelli C, Amati B, Morelli MJ. p53 transcriptional programs in B cells upon exposure to genotoxic stress in vivo: Computational analysis of next-generation sequencing data. *Genomics Data* 2016; **7**: 29–31.

15 Barrett T, Wilhite SE, Ledoux P, Evangelista C, Kim IF, Tomashevsky M *et al.* NCBI GEO: Archive for functional genomics data sets - Update. *Nucleic Acids Res* 2013; **41**:

D991-5.

16 Gray KA, Yates B, Seal RL, Wright MW, Bruford EA. Genenames.org: The HGNC resources in 2015. *Nucleic Acids Res* 2015; **43**: D1079–D1085.

17 Mi H, Huang X, Muruganujan A, Tang H, Mills C, Kang D *et al.* PANTHER version 11: expanded annotation data from Gene Ontology and Reactome pathways, and data analysis tool enhancements. *Nucleic Acids Res* 2017; **45**: D183–D189.

18 Mei S, Qin Q, Wu Q, Sun H, Zheng R, Zang C *et al.* Cistrome Data Browser: a data portal for ChIP-Seq and chromatin accessibility data in human and mouse. *Nucleic Acids Res* 2017; **45**: D658–D662.

19 Fischer M, Grossmann P, Padi M, DeCaprio JA. Integration of TP53, DREAM, MMB-FOXM1 and RB-E2F target gene analyses identifies cell cycle gene regulatory networks. *Nucleic Acids Res* 2016; **44**: 6070–6086.

20 Kuhn RM, Haussler D, Kent WJ. The UCSC genome browser and associated tools. *Brief Bioinform* 2013; **14**: 144–161.

21 Tyner C, Barber GP, Casper J, Clawson H, Diekhans M, Eisenhart C *et al.* The UCSC Genome Browser database: 2017 update. *Nucleic Acids Res* 2017; **45**: D626–D634.

22 Wang S, Sun H, Ma J, Zang C, Wang C, Wang J *et al.* Target analysis by integration of transcriptome and ChIP-seq data with BETA. *Nat Protoc* 2013; **8**: 2502–2515.

23 Liu T, Ortiz JA, Taing L, Meyer CA, Lee B, Zhang Y *et al.* Cistrome: an integrative platform for transcriptional regulation studies. *Genome Biol* 2011; **12**: R83.

24 Fischer M, Quaas M, Steiner L, Engeland K. The p53-p21-DREAM-CDE/CHR pathway regulates G2/M cell cycle genes. *Nucleic Acids Res* 2016; **44**: 164–174.

25 Lickwar CR, Mueller F, Hanlon SE, McNally JG, Lieb JD. Genome-wide protein-DNA binding dynamics suggest a molecular clutch for transcription factor function. *Nature* 2012; **484**: 251–5.

26 Poorey K, Viswanathan R, Carver MN, Karpova TS, Cirimotich SM, McNally JG *et al.* Measuring Chromatin Interaction Dynamics on the Second Time Scale at Single-Copy Genes. *Science* 2013; **342**: 369–372.

27 Baranello L, Kouzine F, Sanford S, Levens D. ChIP bias as a function of cross-linking time. *Chromosom Res* 2016; **24**: 175–181.

28 Quinlan AR, Hall IM. BEDTools: A flexible suite of utilities for comparing genomic features. *Bioinformatics* 2010; **26**: 841–842.

29 Spitzer M, Wildenhain J, Rappsilber J, Tyers M. BoxPlotR: a web tool for generation of box plots. *Nat Methods* 2014; **11**: 121–2.

30 Siepel A, Bejerano G, Pedersen JS, Hinrichs AS, Hou M, Rosenbloom K *et al.* Evolutionarily conserved elements in vertebrate, insect, worm, and yeast genomes. *Genome Res* 2005; **15**: 1034–1050.

31 Heinz S, Benner C, Spann N, Bertolino E, Lin YC, Laslo P *et al.* Simple combinations of lineage-determining transcription factors prime cis-regulatory elements required for macrophage and B cell identities. *Mol Cell* 2010; **38**: 576–89.
