# Supplementary material for: Conservation and divergence of the p53 gene regulatory network between mice and humans
Source: Oncogene. 2019 Feb 1;38(21):4095–109. doi: 10.1038/s41388-019-0706-9 (PMC6755996; doi:10.1038/s41388-019-0706-9)
Supplement: Supplementary file 3 — Supplementary Legends [file 41388_2019_706_MOESM3_ESM.pdf]

Supplementary Table S1

Meta-analysis from 15 genome-wide p53-dependent gene expression profiling data sets for 20,912 mouse genes.

Supplementary Table S2

Primer sequences used for RT- and ChIP-qPCR

Supplementary Table S3

GO term enrichments for particular gene lists identified with the online tool PANTHER v14.0.

Supplementary Table S4

Meta-analysis from 9 genome-wide p53 binding profiles and 7 E2f4 binding profiles for 20,912 mouse genes

Supplementary Table S5

Predicted 636 potential direct p53 target genes in the mouse genome.

Supplementary Table S6

Comparison of the *mouse* and *human p53 Expression Score* for 15,569 one-to-one orthologues.

Supplementary Table S7

1,010 genes predicted to differ in their p53-dependent regulation between mice and humans. These genes display an absolute *p53 Expression Score* ≥ 5 and the absolute difference between the *mouse* and the *human p53 Expression Score* is ≥ 8, and less than three data sets supported their regulation in the other species.

Supplementary Table S8

Meta-analysis from 28 genome-wide p53 binding profiles for 18,845 human genes.
